# A comprehensive framework for integrating lake hypsography and function on a global scale

**DOI:** 10.1038/s44221-025-00461-4

**Published:** 2025-07-17

**Authors:** Cristian Gudasz, Dominic Vachon, Yves T. Prairie

**Affiliations:** 1 Climate Impacts Research Centre, Department of Ecology, Environment and Geoscience, Umeå University, Umeå, Sweden; 2 Département des Sciences Biologiques, Université du Québec à Montréal, Montréal, Québec Canada; 3 Present Address: Ministère de l’Environnement, de la Lutte contre les changements climatiques, de la Faune et des Parcs, Québec, Québec Canada

**Keywords:** Limnology, Macroecology

## Abstract

As climate change and nutrient pollution intensify, understanding how millions of lakes will respond to such forcings as a global or regional collective has become urgent and yet capturing their role in Earthʼs system remain neither conceptually unified nor empirically constrained. Here we introduce a framework that aggregates individual lake hypsography and functional attributes into composite lakes globally, across climate zones or 1-degree Earth system grid cells. We find that globally, lake shape mirrors land rather than ocean, with shallow areas dominating. This structure reveals systematic differences between glaciated and non-glaciated regions and between colder and warmer climate zones. At the 1-degree Earth system grid cells, composite lakes group into five distinct clusters. Globally, an estimated 43% of lake volume and sediment surface area lie within the mixed layer. A composite mixed layer volume-to-sediment-surface-area ratio reveals dominant water column influence and biogeochemical sensitivities, with strong contrasts across climates and glacial histories. The proposed framework advances quantifying and understanding the collective role of lakes across spatial scales in Earthʼs system.

## Abstract (web summary)

This Analysis presents a new framework that integrates lake hypsography and functional attributes at global and climatic scales, revealing key patterns in lake ecosystems and their responses to climate impacts.

## Main

In the most general sense, lakes are water bodies in Earth’s crust that occupy basins formed by diverse geological processes^1^. These transitory landforms, with lifespans ranging from days to millennia, are a testament to the dynamic nature of our planet’s surface. Although advancements in satellite imaging have enhanced our understanding of the global and regional abundance of inland waters^2,3^, a comprehensive description of their depth distribution remains elusive. This gap, particularly in understanding lake hypsography at large scales, is notable. Hypsography is a fundamental concept in aquatic sciences and primarily involves the quantitative analysis of the depth–area relationship, typically represented through histograms. Whereas the hypsography of land and ocean is well established^4,5^, global lake hypsography is lacking, both in terms of detailed individual lake representations and in the form of a consolidated, composite curve comparable to those of land and ocean.

Hypsography directly impacts the physical, chemical and biological responses of lakes to external climate and geochemical forcings^6–8^ and yet it remains understudied, particularly regarding its role in modulating lake responses to climate variability and change^9^. Lake basins can take a wide range of hypsographic shapes, from steep-sided cuvettes to flat basins with a small central depression (Supplementary Fig. 1). Even for lakes of similar mean depth and surface area, contrasting basin shapes strongly determine the relative importance of volumetric and areal processes occurring in the upper and bottom waters of lakes. This is ultimately reflected in rates of whole lake biogeochemical transformation, such as ecosystem metabolism^10,11^, greenhouse gas emissions^12^ and long-term carbon burial rates^7^. The depth distribution also directly impacts biodiversity in the littoral habitat^13^. Lake size and depth are important for the presence and timing of stratification onset and breakup^9,14^. However, the specific regional lake hypsographic characteristics will modulate the response in thermal stratification due to climate or other large-scale perturbations.

Statistical analyses at local, regional and continental scales shed light on fundamental aspects of lake morphometry, such as the power law that relates lake abundance to area^15–17^ and scaling laws linking lake morphometry with biogeochemical functions^6,7,18^. Additionally, multiscale studies highlight variations in ecosystem properties and ecological drivers across different spatial and temporal dimensions^19^. Despite these advances, important questions remain about the effectiveness of statistical models in representing lake functions and their interactions with climate at the continental level. Although focusing on individual lakes provides important insights, it fails to account for emergent properties of biogeochemical and physical dynamics and systemic climate feedback, which only become apparent when analysing aggregated properties and responses from numerous lakes. This limitation implies that the emergent collective behaviours and impacts at regional or continental scales are still not well understood. For example, Earth system models typically operating on 1 × 1 degree grid cells can, at best, integrate lakes by cumulating their surface area within a small number of specific grid tiles^20^. Such aggregation, however, overlooks the well-documented relationship between lake size and greenhouse gas (GHG) emissions. Specifically, numerous small lakes collectively emit disproportionately more CO_2_ and CH_4_ than fewer large lakes of equivalent total surface area^21^. Furthermore, accurately representing the full extent of the littoral area, where much of the CH_4_ emitted from lakes originates, inherently emphasizes the critical importance of small lakes. Consequently, coarse-scale representations based on lake grid tiles may obscure the true contribution of lakes to global GHG fluxes.

Understanding and predicting large-scale phenomena requires developing approaches for the simplification and aggregation of constituent parts appropriate to the scale^22^. This is particularly true when examining individual lake ecosystems, which exhibit variability that may differ from the broader-scale variability observed in climate systems^19^. The challenge lies in establishing causal relationships between phenomena interacting across multiple scales^23^ and their spatial and temporal variation^19^. For example, by aligning our analytical scale to the broader spatio-temporal scales at which climate-driven variations occur, such as by aggregating lake areas by depth to assess the total lake volume or sediment surface area in direct or indirect contact with the atmosphere (that is, above or below the thermocline), we can better discern large-scale spatio-temporal characteristics and dynamics that would otherwise be masked by the inherent variability of individual lakes.

Recognizing these emergent behaviours is vital for large-scale analyses and can expand our traditional understanding of the role of lakes in their landscape. However, to achieve this goal, it is imperative to retain the functional information contained in the hypsography of the individual lakes and aggregate them in such a way that the specific hypsography is preserved. The aggregation of lakes at large spatial scales demands the development of scale-dependent representations that encompass both hypsography and functional responses. These are better suited to reveal emergent properties at different scales, ranging from their sensitivity to climate to their collective role as denitrifiers in agricultural landscapes. Defined as conceptual entities, our approach aggregates depth-specific spatial and functional attributes from a collection of lakes into a unique and scale-specific composite lake representation, akin to a ‘super-’ or ‘Über-lake’. This allows for a comprehensive analysis of emergent properties that manifest at various scales. This collection can be local to a lake district, regional, continental or even global. Due to the hypsographic information of each lake being preserved, the usual lake attributes (for example, mean depth, epilimnetic depth and volume) can be calculated for any composite lake but will differ from the simple average of the individual lakes’ attributes. In this paper, we first describe the underpinning theoretical framework and then, using bathymetric information from >40,000 lakes, develop the tools and models required to estimate the composite lake properties at any scale and in any region of the globe. We then aggregate the individual hypsographies to produce the first illustration of the global lake hypsography and how it differs among climate zones. Lastly, we apply a physical modelling approach to all lakes on Earth to explore and illustrate how the physical attributes of the resulting composite lakes can be used to map and infer functional consequences at larger scales, such as the lake sensitivity to climate.

## Theory

**Fig. 1:**
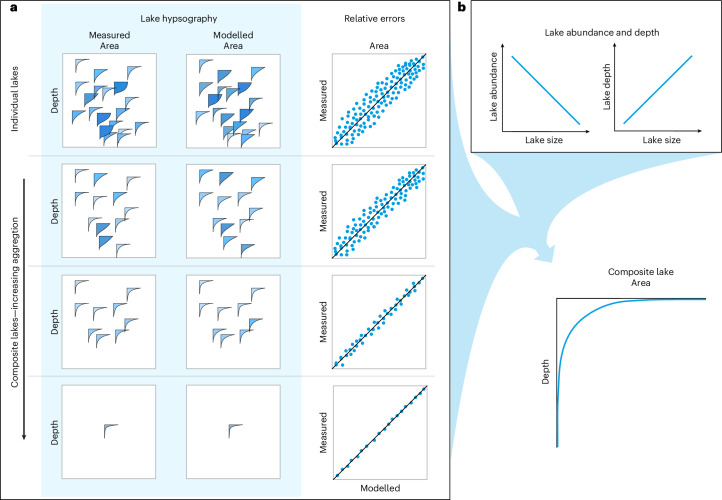
Conceptual diagram illustrating the influence of lake size distribution, depth and relative errors on the composite lake hypsography. **a**, Each icon represents the area–depth of lakes hypsographic curves. The relative errors decrease with increasing aggregation level. The areas and depths alongside comparisons of measured and modelled areas expressed in relative terms. The shaded infill of each hypsographic curve highlights the link between colour and curve shape. **b**, Lake abundance decreases with size follows a Pareto distribution, whereas maximum depth generally increases with lake size; together these factors shape the unique and scale-specific composite hypsography, resulting from the aggregation of areas at depth across lakes in a collection.

The hypsographic shape of a composite lake at any scale or region ultimately results from the interplay between the Pareto law governing lake size distribution^15,16^ and the particular relationship linking depth with lake size applicable at that scale^18,24^. This dictates the distribution of the area and volume across various depths. As a result, the abundance of small and shallow lakes can accentuate the flatter portion of the composite lake hypsographic curve. This relationship underscores the substantial influence of the lake size distribution on the overall structure of the shape of the hypsographic curve (Fig. 1). Hence, to elucidate the shape of the composite lakes hypsography within a region, we must quantify the interplay of two primary factors: the distribution of lake size abundance^15–17^ and the relationship between maximum depth (*Z*_max_) and lake area. Individual lakes invariably show a monotonic decrease in area with increasing depth, a feature that applies to the composite lake hypsography. In this context, *Z*_max_ becomes a crucial factor to determine how much area of each lake contributes to the overall hypsography at a given depth. Although the exact scaling of *Z*_max_ with lake area exhibits considerable variability, both empirical observations^25–29^ and mechanistic models^18^ have shown a generally increasing trend of *Z*_max_ with lake size.

Lakes globally span a wide range of basin shapes, and accurately representing this diversity through a simplified, yet accurate hypsographic description presents a major challenge. Similarly, it is essential to understand how errors in predicting lake morphometry parameters for individual lakes can collectively influence and propagate inaccuracies in the composite lake hypsography. Although the composite lake hypsography aggregates the area at each depth from all lakes in a specific region, the errors associated with the modelling individual lake hypsographies may not accumulate at the same rate as area does. When aggregating modelled areas, their respective errors also accumulate, leading to an increase in the total variance. However, the relative error of the aggregated area at depth defined as the ratio of modelling error to aggregate measured area decreases. Consequently, the precision of the aggregated area improves. When aggregating more lakes, area increases at a faster rate than errors do. The rate at which area and errors increase in the composite is influenced by the specific distribution of lake sizes and maximum depth used in aggregation. Within the composite hypsographic curve, deeper areas are likely to exhibit greater errors due to the aggregation of fewer lakes. This pattern reflects the Pareto distribution, where the abundance of lakes decreases with increasing size. From a statistical point of view, the relative error of individual lake physical properties (that is, hypsography, lake shape) can be reduced through aggregation of areas at depth across lakes. When aggregating areas at each depth from a sufficiently large number of lake hypsographies characterized by a certain degree of error, these discrepancies could compensate for each other, leading to a relatively stable overall result (Fig. 1). As a result, the composite lake hypsography uncertainty may be better constrained (albeit subject to the particular error structure) and should be construed as a unique property of the aggregated data.

## Results and discussion

### The global lake

**Fig. 2:**
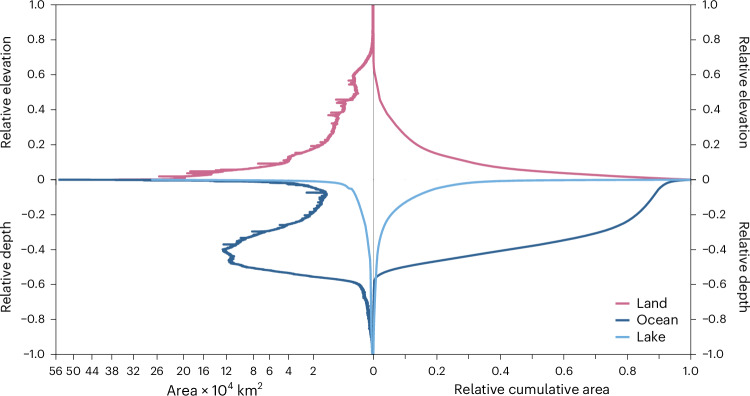
Comparison of global landforms hypsography. The global hypsography of land, lake and ocean; the relative area is represented on a square root scale for better visualization. Data for present day land and ocean hypsography based on ETOPO1 global relief model (accessed 2021)^4,5^.

The global composite lake hypsography constructed through the aggregation of the modelled individual hypsography of all 5,735,587 lakes larger than 1 ha (Supplementary Fig. 2) exhibited an area of 2,408,550 km^2^, a volume of 163,117 km^3^, and a mean depth of 67.7 m, respectively. The maximum depth of 1,632 m corresponds to that of Lake Baikal. The global land hypsography curve shows a rapidly decreasing area with elevation with a steep decrease corresponding to the mountains and a flattened base corresponding to the plains. Unlike the land, global ocean hypsography (Fig. 2) is primarily influenced by the large area of the Abyssal Plains, resembling a deep dish with a sharp increase in depth corresponding to the deep trenches. The shape of the hypsographic curve of the global lake instead closely resembles that of the inverted global land (Fig. 2). It highlights the predominant influence of the contribution of shallow areas dictated by the governing Pareto lakes size and depth distribution. As a result, the abundance of small and shallow lakes accentuates the flatter portion of the global lake hypsographic curve. This is evidenced by the high dynamic ratio (DR_C_), defined as the ratio of the square root of lake area to mean depth, indicating the influence of aggregated areas from shallow lakes, as detailed in Table 1. The analysis of uncertainties in modelling lake hypsography (Methods, Supplementary Methods and Supplementary Text) showed that the relative errors of areas and volumes at various depths decrease substantially from individual to composite lakes. This reduced relative uncertainty suggests an emergent property of the aggregation, probably due to multiple factors: individual random errors may cancel out, increased sample sizes across lakes and depths facilitate outlier smoothing and a scale effect may reduce the influence of local variations, allowing more stable and predictable broad patterns to emerge. A similar behaviour was observed where the modelled lake volumes were an order of magnitude larger in individual, compared to the aggregated lake volumes^24^.

**Table 1:** The global and regional composite lake parameters describing morphometry and functionality

Parameter	Global	Glaciated	N-G	Polar	Cold	Temp.	Arid	Tropical
Lake #	5,735,587	3,683,960	2,051,627	1,152,386	3,961,100	268,365	154,292	199,444
SA_C_ (km^2^)	2,408,550	1,154,794	1,253,756	215,321	1,260,197	89,588	584,358	259,085
DR_C_	22.9	44.8	10.4	30.3	28.1	9.1	6.1	3.9
*Z*_maxC_ (m)	1,632	836	1,632	836	1,632	438	1,025	1,471
*Z*_meanC_ (m)	67.7	24.0	108.0	15.3	40.0	33.0	124.8	129.6
wT_meanC_	11.0	6.0	12.0	5.9	4.9	12.7	10.0	22.6
*V*_C_ (km^3^)	163,117	27,680	135,438	3,295	50,366	2,954	72,917	33,585
fV_EpiC_	0.43	0.51	0.41	0.38	0.44	0.44	0.51	0.23
fA_EpiC_	0.43	0.34	0.51	0.21	0.35	0.71	0.55	0.59
*V*_EpiC _/ *A*_EpiC_ (m^3^ m^−^^2^)	67.8	36.1	87.5	27.1	50.0	20.3	116.1	50.0

Lake numbers (#), surface area (SA_C_), dynamic ratio (DR_C_), maximum depth (*Z*_maxC_), mean depth (*Z*_meanC_), vertically resolved mean water temperature when weighted by volume (wT_meanC_), volume (*V*_C_), fraction of epilimnetic volume (fV_EpiC_), fraction of epilimnetic sediment surface area (fA_EpiC_), ratio of epilimnetic volume to epilimnetic sediment surface area (*V*_EpiC _/ *A*_EpiC_). Throughout this table, N-G refers to non-glaciated and Temp. to temperate regions, and ‘_C_’ refers to composite value across the parameters.

Physical dynamics are a defining characteristic of lake functioning. They reflect how climate forcings interact with a body of water and its hypsography. The resulting thermal structure and mixing regimes determine how lake pelagic and benthic habitats (that is, water volumes and sediment surface areas) are partitioned and are key variables governing ecological and biogeochemical processes^30^. Through the systematic application of a physical dynamics model to all lakes, we estimated the composite fraction of epilimnetic volume (fV_EpiC_) and epilimnetic sediment surface area (fA_EpiC_) and vertically resolved mean water volume-weighted mean water temperature (wT_meanC_) (Methods). We estimated that globally, only 43% of sediment surface area and water volume are within the mixed surface layer, that is, in contact with the atmosphere year round and a mean water temperature of 11 °C, indicating a generally cold global lake, reflecting the distribution of most lake areas and volume in cold regions.

The mean depth of the composite global lake (*Z*_meanC_), defined as the ratio of composite water volume (*V*_C_) to composite lake surface area (SA_C_), can be viewed as a metric describing the overall relative importance of sediment vs water processes. Simple yet foundational lake metrics, rooted in first principles such as area and volume could serve as a starting point for identifying patterns at regional to global scales. We used the terms area-based and volume-based processes to emphasize the importance of sediment surface area versus water volume in scaling ecological processes in lakes. In its simplest form, area-based or volume-based processes refer to the dimensions that limit the magnitude of a flux at the ecosystem level. Area-based processes typically involve interactions either at the lake bottom sediment–water interface (for example, benthic respiration, nutrient flux from sediments, benthic primary production) or at the air–water interface (gas exchange with the atmosphere, incoming solar radiation). These processes scale with the benthic and surface area of the lake, respectively. In contrast, volume-based processes occur within the water column (for example, pelagic respiration, planktonic nutrient cycling, heat storage) and therefore scale with the volume of water.

The relative strength of the water column–sediment interactions has implications for controlling lake metabolism, nutrient cycling and greenhouse gas sources and dynamics and is reflected by the ratio of epilimnetic volume to sediment surface area^10,31^. By examining this ratio in composite lakes, we can uncover region-wide patterns, thresholds or controlling factors (for example, susceptibility to anoxia or nutrient cycling shifts) that remain hidden when analysing lakes individually. Using the thermal stratification and seasonal mixing regimes of lakes, we calculated in a similar way (Methods) the ratio of the composite epilimnetic volume (*V*_EpiC_) to epilimnetic sediment surface area (*A*_EpiC_) of the global lake. This can be viewed as a functional index constraining the importance of area- vs volume-based processes as it pertains to lake–atmosphere exchange, and it can differ markedly from *V*_C _/ SA_C_ (or *Z*_meanC_). Therefore, the *V*_EpiC _/ *A*_EpiC_ ratio provides a simple metric of the dominant pathways that are likely to be under direct climate influence.

In our analysis, both *V*_C _/ SA_C_ and the estimated global *V*_EpiC _/ *A*_EpiC_ were similar, with a value of approximately 68. To compare the contribution of sediment-based processes to those in the water column on a per-area basis, we calculated the average volume of epilimnetic water above each square metre of the sediment surface area by multiplying the fraction of epilimnetic volume fA_EpiC_ by the *V*_EpiC _/ *A*_EpiC_ ratio. Values below and above the threshold of 68 imply dominance of the water column and sediment in the epilimnion, respectively. In other words, for each square metre of lake surface, there are approximately 29 m^3^ of epilimnetic water. Furthermore, the estimated fA_EpiC_ suggests that for each square metre of lake surface, 0.43 m^2^ is represented by epilimnetic sediments. Hence, on the global scale, the rate of any area-based process in the epilimnetic sediments must be 68 times higher on an area basis to match the influence of a comparable volume-based process in the water column. As a first-order approximation, the ratio of the average respiration rate of water column (15.7 mmol O_2_ m^−3^ d^−1^) to sediment (10.3 mmol O_2_ m^−2^ d^−1^) of 1.5 (ref. ^32^) would suggest that the global contribution of respired CO_2_ in the water column is much higher than that of the sediments. Whereas the *V*_EpiC _/ *A*_EpiC_ ratio represents a metric for assessing composite lakes functionality at continental scales, its implications remain to be fully explored, and future work is needed.

### The climate regions lakes

When aggregating the hypsography of lakes across regions, it is essential to focus on the patterns emerging within specific climate regions. The relationship between hypsographic shape and lake origin is complex; lakes of the same origin can vary substantially in shape and often overlap in characteristics with lakes of different origins^33^. Factors such as climate, relief, lithology and lake drainage size all play a role in the balance between sediment accumulation and denudation, reshaping the original lake hypsography^34^. Region-wide fluctuations in lake water levels, linked to contemporary climate, primarily affect smaller lakes, which respond rapidly and dynamically thereby altering the overall lake size distribution^35^. In addition to influencing water availability, climate affects various physical lake processes, including the heat budget and turbulent mixing. Consequently, a climate-focused analysis of lake hypsography, as opposed to origin, is instrumental in revealing patterns crucial for understanding the macroecology of lakes.

**Fig. 3:**
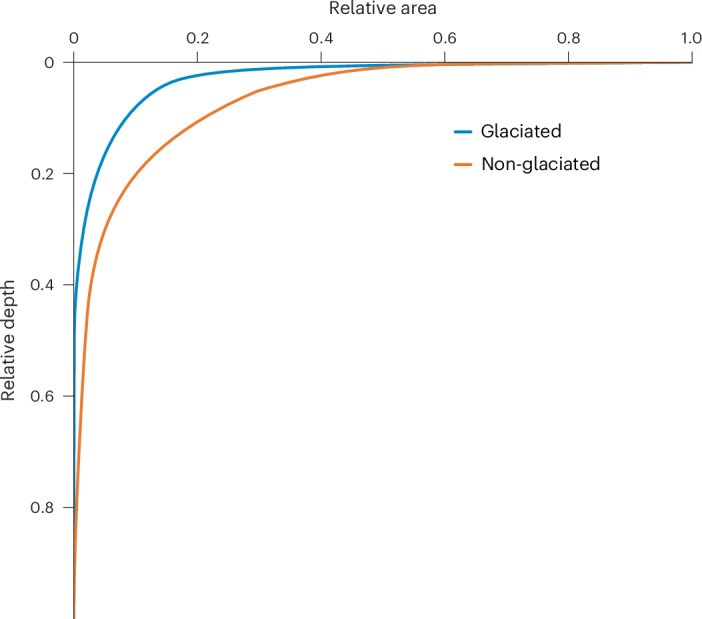
The glaciated and non-glaciated regionsʼ lake hypsography. This graph shows the glaciated and non-glaciated regionsʼ composite lake hypsography.

Climate and its associated hydrological regimes are one of the most important variables regulating the extent and persistence of lake surfaces and their functioning. Historically, the Quaternary glaciation with alternating glacial and interglacial periods also had a substantial impact on the formation and destruction of lakes, affecting lake size distribution^36,37^ and consequently global lake hypsography. The effects of the last ice retreat as delimited by the Last Glacial Maximum^38^, have led to the formation of the highest lake density regions on Earth, resulting in about twice as many lakes in the glaciated areas compared to the non-glaciated areas of the globe (Table 1). This reflected the difference between the glaciated and non-glaciated composite lakes hypsography and their properties (Fig. 3 and Table 1). Although the composite lake area is approximately similar in the glaciated and non-glaciated regions, there is 4.9 times more volume in the non-glaciated region. The *Z*_meanC_ is quite distinct between the glaciated and non-glaciated regions (24 and 108 m), showing the higher contributions of the area vs volume between the regions. Regional differences in the lake size and depth distribution between glaciated versus non-glaciated regions influence the shape of the composite lake hypsographic curve (Fig. 3). This is evidenced by a marked increase in the DR_C_, which rose from 10.4 in non-glaciated to 44.8 in glaciated regions. The fundamental cause of this disparity is the greater prevalence of numerous small and shallow lakes in glaciated areas. Although glaciated and non-glaciated regions exhibit similar Pareto slopes (−0.98 and −0.92, respectively), indicating a comparable distribution pattern of lake sizes, the glaciated region had increased smaller and shallower lakes by 64%. The interaction between climate, lake physics and hypsography resulted in an increase in the relative importance of the epilimnetic sediment surface area and volume in the non-glaciated compared to glaciated regions composite lakes. Whereas the fV_EpiC_ is approximately similar, 0.51 and 0.41, respectively, the epilimnetic volume is about four times larger in the non-glaciated region. A low fA_EpiC_ in the glaciated (0.34) compared to the non-glaciated region (0.51) results in 1.6 times higher epilimnetic sediment surface area in the latter.

**Fig. 4:**
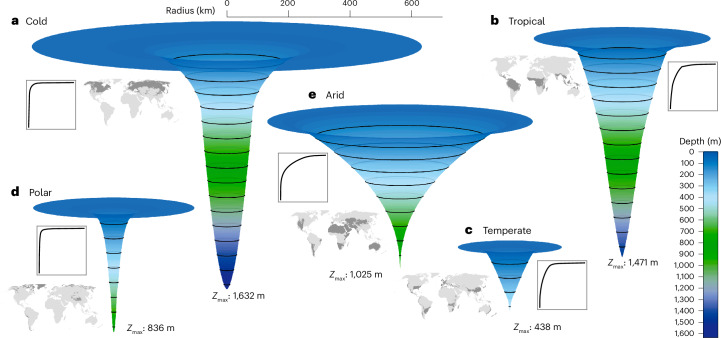
The climate zones composite lakes hypsography. The 3D circular representation was created by revolving the area at depth profile (as circular cross-sections, with radius = √area, scaled to km) around the vertical depth axis of the composite lakes of the main climate zones (left panel maps) based on the present Köppen–Geiger climate classification^39^. **a**–**e**, The sub-climate zones were aggregated to reflect the Cold (**a**), Tropical (**b**), Temperate (**c**), Polar (**d**) and Arid (**e**) climates. The composite lakes scale is absolute in km. The dark grey on the global maps represents the area of the climate zone coverage^39^. The 2D graphs show the equivalent hypsographic curve with relative depth (*x* axis) and relative area at depth (*y* axis) on a 1:1 scale.

The aggregation of individual lakes areas at depth within the main Köppen–Geiger climate regions^39^ has unveiled distinct and contrasting composite hypsographies (Fig. 4 and Table 1). Specifically, we identified two primary types of composite lake hypsography: the Cold and Polar region shows a spindle shape with an expansive flattened top (Fig. 4a,d), whereas Tropical, Temperate and Arid regions show a reduced shallow sediment surface area contribution, dominated instead by larger and deeper lakes (Fig. 4b,c,e). Such shape differences among climate regions can be partly attributed to the number of large lakes in each zone, but a comparison of regions with similar large and deep lakes (for example, Tropical vs Cold) show distinctive hypsographies. This dichotomy is further reflected in the patterns of epilimnetic volumes and sediment surface areas and their respective ratios, highlighting underlying physical dynamics and hypsographic distinctions. In our analysis, the fraction of epilimnetic volume (fV_EpiC_) varied from 0.23 to 0.51, indicating a general trend toward lower epilimnetic volume fractions, with notable exceptions in Cold, Temperate and Arid regions (Table 1). Regarding fA_EpiC_, the Arid, Temperate and Tropical regions reported substantially higher values of 0.55, 0.71 and 0.59, respectively, compared to the Polar and Cold regions, which had lower fractions of 0.21 and 0.35.

The *V*_EpiC_/*A*_EpiC_ ratio, which represents the volume-weighted mixed layer depth, distinctly reflects regional disparities, as shown in Table 1. In this regard, the Temperate and Arid regions display the most pronounced contrasts, with ratios of 20.3 and 116.1, respectively. These values emphasize the predominant contribution of epilimnetic sediment surface areas in the Temperate region compared to water volumes in the Arid region. Specifically, in the Polar, Temperate and Tropical regions, the amount of epilimnetic water per square metre of lake surface is 5.8, 14.5 and 29.5 m^3^ (calculated as fA_EpiC_ × (*V*_EpiC_/*A*_EpiC_)), and the corresponding epilimnetic sediment surface areas (fA_EpiC_) are 0.21, 0.71 and 0.59 m^2^, respectively. This disparity indicates that for any area-based process rate occurring in the epilimnetic sediments to have an equivalent influence as a volume-based process in the epilimnetic water column, it must be 27.1, 20.3 and 50.0 times more effective, respectively, on an area basis. In contrast, the Cold and Arid regions, with their increased epilimnetic volumes, show for each square metre of the sediment surface, 17.7 and 64.0 m^3^ of epilimnetic water and 0.35 and 0.55 m^2^ of epilimnetic sediment, respectively. This results in a substantially higher scaling factor for epilimnetic sediment process rates, specifically 50.0 and 116. times, respectively, to match the influence of process rates in the water column. These contrasts reflect not only geographic or climate variability but also potentially divergent ecological and biogeochemical processes, such as dissolved oxygen and methane dynamics. For example, a larger proportion of the sediment surface area in direct contact with the atmosphere through the epilimnetic waters translates into a strong response to methane production^12^. Similarly, a larger proportion of volume out of direct contact with the atmosphere leads to increased oxygen depletion^40^.

### 1-by-1-degree grid lakes

**Fig. 5:**
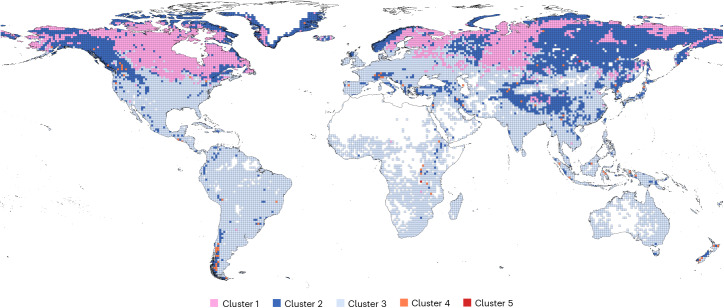
Map of the global clusters of composite lakes organized by 1-by-1-degree grid cells. Further information regarding the cluster analysis is available in Methods and Supplementary Table 4.

The variability in climate across different regions and the operational scales of Earth system models, typically at a 1 × 1 degree spatial scale^41^, underscore the importance of understanding lake characteristics at corresponding scales. To align with these climate interaction scales, we aggregated area from individual lake hypsographies at the 1-degree grid level. This approach not only captures spatial patterns in lake abundance but also reveals distinct geographic clusters of composite lakes (Fig. 5 and Supplementary Table 4), offering valuable insights into regional lake dynamics and their potential sensitivity to climate.

The analysis, conducted on composite lakes organized by 1 × 1 degree grid cells (Methods), identified five clusters with distinct characteristics as detailed in Supplementary Table 4 by their median values. Cluster 2, which comprised 2,619 grid cells, contained 1,023 lakes per cell, the highest density among all clusters. This cluster was predominantly located in cold climates with high lake densities, such as North America, Scandinavia, Siberia and the Tibetan Plateau. It features a substantial surface area of 214.5 km^2^, a relatively low volume of 0.62 km^3^ and shallow *Z*_max_ and *Z*_mean_ of 17 m and 2.8 m, respectively. This cluster had the highest DR_C_ of all clusters, at 4.7, indicating a larger contribution from the aggregation of lake areas from shallow lakes. Cluster 1, with 4,021 grids and 80 lakes, featured smaller lakes with an area of 9.5 km^2^ and a low volume of 0.05 km^3^, alongside slightly deeper *Z*_max_ and *Z*_mean_ depths of 25.1 m and 4.9 m. Its DR_C_ of 0.5 suggested decreased contributions from shallow areas compared to Cluster 2. Cluster 3, encompassing 7,753 grids, included 33 lakes with the smallest surface area of 4.4 km^2^ and a volume of 0.01 km^3^ and depths similar to Cluster 1, reflected in a low DR_C_ of 0.6, comparable to Cluster 2. Cluster 4 occupied fewer grids (82) but contained 89.5 lakes with a substantial area of 340.2 km^2^, a large volume of 32.53 km^3^ and substantially larger *Z*_max_ and *Z*_mean_ depths of 306.0 m and 105.8 m, resulting in a very low DR_C_ of 0.2. Finally, Cluster 5 stood out with only 11.5 lakes across two grids, aggregating the largest lakes with the most extensive areas and volumes, alongside the deepest *Z*_max_ and *Z*_mean_, maintaining a similarly low DR_C_ to Clusters 1, 3 and 4.

**Fig. 6:**
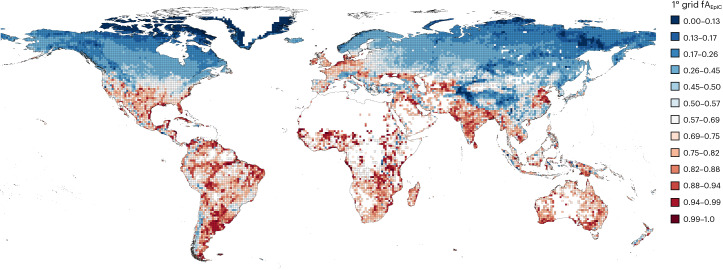
Grid-based functional properties of composite lakes. The proportion of epilimnetic sediment surface area of composite lakes, organized by 1 × 1 degree grid cells as used in climate models, during 2018.

Grid-level analysis fV_EpiC_ and fA_EpiC_ showed values below 31% for most clusters, except for Cluster 3 (Fig. 6 and Supplementary Table 4). Around 45° N latitude, grid-level fA_EpiC_ data indicated a division into two groups at a 60% epilimnetic sediment surface area threshold. Grids below this latitude exhibited higher fA_EpiC_ values, with extremes at the lowest and highest latitudes. Clusters 1–3 had a median *V*_EpiC_/*A*_EpiC_ ratio between 3 and 5.7, suggesting an increased contribution of the epilimnetic sediment surface area. Conversely, clusters 4 and 5, dominated by larger lakes, showed a volume-dominated *V*_EpiC_/*A*_EpiC_ ratio of 108.1 and 673, respectively. For clusters 1–3, the median amount of epilimnetic water per square metre of lake surface ranged from 1.2, 0.9 and 2.2 m^3^ fA_EpiC_ × (*V*_EpiC_/*A*_EpiC_), and the corresponding epilimnetic sediment surface areas were 0.23, 0.29 and 0.74 m^2^ (fA_EpiC_). This indicates that area-based process rates in the epilimnetic sediments must be 5.7, 3.0 and 3.0 times more effective on an area basis to match the volume-based processes rates in the epilimnetic water column. It suggests that at this scale, the gap between water column and sediment process rates is much smaller, which potentially can be more easily altered in response to climate. In clusters 4 and 5, the larger epilimnetic volumes per square metre of lake surface resulted in 33.1 and 174.3 m^3^ of epilimnetic water and 0.31 and 0.25 m^2^ of epilimnetic sediment. This required a substantially higher scaling factor for sediment processes rates—108.1 and 673.0 times, respectively—to equal the impact of processes in the epilimnetic water column.

### Advances and implications for lake ecosystem science

Our ability to predict the causes and consequences of global climate change hinges on effectively bridging processes across various spatial and temporal scales^22^. This is particularly true for lake ecosystems. Analysing a population of lakes as a distribution emphasizes the unique responses and characteristics of each lake within the collective, highlighting individual variability and patterns. In contrast, focusing on lakes through the lens of functional aggregates centres on the collective behaviour and shared properties of the lake population. Aggregating properties and responses of multiple lakes not only facilitates the understanding of broader ecological dynamics but also potentially revealing emergent properties not apparent when analysing lakes individually. Aggregating lake-specific characteristics and retaining their unique hypsographic features and the associated physical and biogeochemical dynamics allows us to reevaluate this scaling at continental and global levels. The concept of planetary boundaries has been emerging to quantify the processes that regulate the stability and resilience of Earth’s system^42–44^. Carpenter and Bennett^45^, using a simple generic representation of global freshwaters, examined the planetary boundary of phosphorus and its consequences. By employing metrics derived from composite lakes, such as the volume to area ratio, predictor of dissolved oxygen depletion rates in lakes^46^, we may identify region-wide thresholds, trends and controlling factors related to oxygen depletion driven by phosphorus dynamics that would remain undetected when examining individual lake systems in isolation. This comprehensive framework introduces capabilities to quantify the stability and resilience of global freshwater systems and planetary boundaries. Nevertheless, these findings underscore the importance of further research that translate into accurate, quantitative predictions of regional and global lake ecosystems behaviour, while addressing remaining uncertainties to maximize its applicability and robustness.

The framework developed here serves as a lens through which to view and decode the biogeochemical functions and dynamics of lakes at broad spatial scales, bridging the gap between regional drivers and local lake characteristics that influence specific functional responses, such as the carbon cycle^47^. Moreover, it opens avenues to address pivotal questions regarding the role of lakes at Earth system scales, potentially revealing emergent properties and redefining the conceptual framework of lake macroecology.

## Methods

The methods used throughout this study can be generally described on four distinct levels: (1) global lake map and hypsography, (2) individual lake physical modelling, (3) composite lake level analyses and (4) evaluating and propagating uncertainties.

### Global lake map and hypsography

An overview of the methods and analyses used to construct the hypsography of composite lakes are detailed in Supplementary Fig. 2. Our general approach consisted of a sequence of five steps:

1. Compilation of a global lake morphometry dataset (*Z*_mean_, *Z*_max_ and lake area) and lake bathymetric dataset to generate an updated global lake map.
2. Development and validation of predictive models using machine learning algorithms to estimate lake hypsography parameters (*Z*_mean_, *Z*_max_ and shape parameter *q*, described below) from readily available topographic features of the surrounding landscapes.
3. Validation of a general hypsographic model for lakes.
4. Application of the models to all 5.74 million lakes on Earth greater than 1 ha.
5. Aggregation of the lake hypsography at various regional and global scales (described in detail in the composite lake level analyses section).

### Lake morphometry data

To develop a bathymetric model applicable worldwide, we compiled a global lake dataset of 39,808 lakes with lake morphometry data with geographical location and a minimum set of variables necessary to apply a generic but highly flexible hypsography model (below) such as *Z*_max_, *Z*_mean_ and lake surface area (SA). A summary of these data is presented in Supplementary Table 1. Although our compilation of lake morphometry data is extensive, it is likely that the data quality varies between individual sources and this was not formally assessed. This should be considered when interpreting the results of our analysis. The dataset encompasses a broad spectrum of values and geographical regions. We then evaluated the distribution of lakes across various major landform categories, comparing it with the global lake data (Supplementary Fig. 3b,c). Our analysis indicated a well-represented range of major landforms such as mountains, plains, lowlands, plateaus and hills. These classifications were derived from the global landforms^48^ and used 0.5° rasters^49^. Each lake’s coordinates were mapped to these raster cells to categorize them accordingly using the 0.5° raster data. For climate zoning, we utilized the Koppen–Geiger climate zones^39^. We aggregated the climate subgroups into the primary categories of Arid, Cold, Polar, Temperate and Tropical. Lakes were assigned to regions based on the coordinates of their polygon centroids, which were matched to the corresponding raster cells defining each climate zone. We then calculated the proportion of lakes in each major climate region as follows: Arid (0.52%), Cold (95.10%), Polar (0.40%), Temperate (3.77%) and Tropical (0.22%). Relevant data sources are listed in Supplementary Table 2.

### Global lake map

Our study utilized the 2018 Global Surface Water (GSW) mapping^3^ from which we removed reservoirs to create a global lake map (GSWL) of natural lakes larger than 1 ha. Details of our approach can be found in Supplementary Methods.

### Modelling individual lake hypsography

A lake hypsography model that can be applied globally must accommodate a large diversity of lake basin shapes. Imboden^50^ described a simple lake hypsography model that uses two parameters such as maximum (*Z*_max_) depth and an exponent *q* the general 2D shape of a lake (equation (1)).

1 $${A}_{z}=\mathrm{SA}{\left(1-Z/{Z}_{\max }\right)}^{q}$$

Or, in relative terms:

2 $${A}_{z,\mathrm{rel}}={\left(1-Z/{Z}_{\max }\right)}^{q}$$

where *A*_*Z*_ is the planar area (m^2^) at depth *Z* (m), is the relative area at depth, SA is the lake’s surface area (m^2^), *Z*_max_ is the maximum depth (m) and *q* is a dimensionless exponent describing the general 2D shape of the lake (that is, relative area at depth). A *q* value of 2 corresponds to a perfect cone, whereas values below or above this threshold represent more convex or concave forms, respectively. The Imboden model can therefore accommodate a wide suite of hypsographic shapes (depicted in Supplementary Fig. 1), and while its *q* parameter is best estimated from measured lake mean and maximum depths of the morphometry dataset as:

3 $$q=\frac{{Z}_{\max }}{{Z}_{\mathrm{mean}}}-1$$

Assuming the general adequacy of the lake hypsography model (equation (1)), it can be used to easily derive other useful aspects of lake hypsography, which were needed for the individual lake physical and biogeochemical modelling and the composite lakes construction. The total lake volume (m^3^) can be calculated as:

4 $$V=\mathrm{SA}\left(\frac{{Z}_{\max }}{q+1}\right)$$

and the volume below a given depth (m^3^) as:

5 $${V}_{z}=\mathrm{SA}\left(\frac{\left({Z}_{\max }-Z\right){\left(1-\frac{Z}{{Z}_{\max }}\right)}^{q}}{q+1}\right)$$

In vertically discretized lake representations, equations (1) and (4) are used to calculate the differential area (d*A*_*Z*_) and water volume (d*V*_*Z*_) between two consecutive depths (*Z* and *Z* + *1)* as:

6 $${{\mathrm{d}A}}_{z}={A}_{Z}-{A}_{Z+1}$$

7 $${{\mathrm{d}V}}_{z}={V}_{z}-{V}_{z+1}$$

### Predicting lake morphometry at global scale

We used the morphometry dataset of 39,808 lakes to build predictive models of *Z*_max_, *Z*_mean_ and *q* based on the lake surrounding topographic features to apply to the global lake map. We used global rasters at 250-m resolution GMTED2010 of topography variable such as elevation, slope, roughness, terrain roughness index, topographic position index, profile curvature and tangential curvature^51^. We employed buffers, of 20, 40, 80, 160, 320 and 640 percent of the circular equivalent radius of each lake polygon, to analyse the impact of surrounding topography on lake morphometry. This range was selected to effectively capture landscape variability at various spatial scales, aiding in the identification of the topographical extent most relevant to lake morphometric variations. The area of the lakes was removed in each of the topographic raster before analysis. We then applied zonal statistics to the topographic rasters surrounding each lake to calculate the mean and standard deviation (SD) of the topography within each buffer zone using the open-source QGIS geographic information system^52^. These statistical measures were then utilized as input variables in our predictive models to random forest algorithms predicting mean depth, maximum depth and *q* parameter. The DEM resolution can impact landscape detail capture, and whereas our predictive model utilizes mean and SD across topography and buffers, the 250-m resolution may decrease variability and is potentially limiting in flat landscapes. We note that there was a mismatch in the time between the GSW and GMTED2010 datasets of 2018 and 2000, respectively.

Initial model development was obtained after comparing the performance of several machine learning algorithms (random forests, boosted gradients, artificial neural networks) using Akaike’s information criterion. This comparison indicated that the random forest algorithm performed best both in the model training and model validation stages, with a random 20% of observations reserved for validation. However, analysis of the residuals suggested that the buffer widths useful in the prediction changed, unsurprisingly, with lake sizes, such as 160, 320, 640 percent buffers for lakes <10 km^2^ and 20, 40 and 80 percent buffers for lakes >10 km^2^. We thus divided our dataset into small (0.002 km^2 ^< lake area < 10 km^2^) and large ( > 10 km^2^) lakes and developed separate models of *Z*_max_, *Z*_mean_ and *q* (the latter parameter describes the shape of lake and is explained in the next section) for each of the lake size categories (Supplementary Fig. 4a–c). In our predictive modelling of *Z*_max_, *Z*_mean_ and *q*, we encountered a non-normal distribution of prediction errors. To address this, we applied a cube root (power of 0.33) transformation to the lake morphometric variables, that is, the dependent data used in the model. This transformation was effective in stabilizing the variance and achieving a distribution of errors (in the transformed space) that was closer to normality, thereby enhancing the overall goodness of fit for our models and simplifying uncertainty quantification. The observed vs estimated of *Z*_max_, *Z*_mean_ and *q* parameters for both small and large lakes can be found in Supplementary Fig. 4a–c. The summary of the models’ goodness of fit can be found in Supplementary Table 3. We applied the predictive models to the global lake map to predict *Z*_max_, *Z*_mean_ and *q* for each lake of the GSWL dataset. We used the original *Z*_max_, *Z*_mean_ and *q* values from 115 of the 243 large lakes ( > 400 km^2^) in the final GSWL dataset to reduce prediction error.

### Individual physical lake modelling

Lakes globally show a wide range of physico-chemical properties. Such a diversity of lake ecosystems and conditions and their coupling to functional responses is difficult to capture in models at global scales, primarily due to the lack of essential data to parameterize the physical or biogeochemical models (that is, no good data exists on critical parameters that are important for lake energy balance as simple as water transparency).

Out of the 5,784,180 lakes in GSWL dataset, 48,593 located along the continental shorelines were excluded due to the lack of climate forcing data. This was caused by the placement of the 0.1/0.1° grids of the fifth-generation global reanalysis produced by the European Centre for Medium-Range Weather Forecasts (ERA5, by ECMWF)-Land data along the shorelines. To keep a consistent analysis throughout, we report a global dataset of 5,735,587 lakes. We used Simstrat v. 3.01^53^, a one-dimensional physical lake model for the simulation of stratification and mixing, which describes turbulent mixing including energy transfer to internal seiches. It has been successfully applied to lakes with different morphometries^54–56^ and climate^14,57^ (Supplementary Methods). Simstrat also estimates the ice build-up in lakes in cold climates, with important consequences on gas dynamics. Further details in Supplementary Methods.

The thermocline depth was determined as the shallowest depth at which the water density change exceeded 0.1 kg m^−4^ (ref. ^58^). If the maximum density change was equal or less than 0.1 kg m^−^^4^, a thermocline equal to maximum depth was assigned (that is, water column fully mixed without any thermocline). On this basis, we derived daily average thermocline depth (dZ_therm_*)*. We used also used the Simstrat output daily ice thickness (dIce) to derive the duration of the ice cover. The annual mean temperature profile (annually integrated value for each depth; *T*_meanZ_) for individual lakes was derived based on hydrodynamic model output and thermocline depth calculations. We then calculated the fraction of the time over one year that a given depth (*Z*) is isolated from direct contact with the atmosphere (fIso_atmZ_), that is, either under thermocline or under ice cover condition. This ranges from 0 (never isolated) to 1 (isolated all year long).

### Composite lake level analyses

The composite lake construction is done through the depth-by-depth aggregation of lake characteristics. We aggregated areas at depth from modelled hypsography and functional features such as epilimnetic sediment surface areas and volumes of all lakes to create composite hypsography of the global lake and various regional subsets. For the climate region lakes, we used the main climate zones based on the current Köppen–Geiger climate zones^39^. The vector map of the Last Glacial Maximum^38^ was used to classify lakes and create the glaciated and non-glaciated composite lakes. We developed 1-degree grids to simulate lakes at spatial scales representative for Earth system models. These grids were then utilized to create composite lake representations.

#### Calculating the composite lakes hypsography characteristics

For each composite lake, we derived a suite of parameters describing morphometry and functionality. We calculated composite lake areas and volumes at specific depths using a generalized hypsographic model, summing the contributions from individual lakes (*i*) across all lakes (*n*), as described below.

Total surface lake area:

8 $${\mathrm{SA}}_\mathrm{C}=\mathop{\sum}\limits_{i=1}^{i=n}{\mathrm{SA}}_{i}$$

Represents the combined surface lake area.

Lake area at depth (*A*_C*,z*_):

9 $${A}_{\mathrm{C},z}=\mathop{\sum}\limits_{i=1}^{i=n}{\mathrm{SA}}_{i}{\left(1-\frac{Z}{{Z}_{\max ,i}}\right)}^{{q}_{i}}$$

Represents the combined lake area at depth *Z*.

Differential area at depth (d*A*_C*,z*_):

10 $${{\mathrm{d}A}}_{{\rm{C}},{{z}}}=\mathop{\sum}\limits_{i=1}^{i=n}{{\mathrm{d}A}}_{z,i}$$

Represents the combined incremental change in lake area between two consecutive depths (*Z* and Z + 1*)*.

Total volume (*V*_C_):

11 $${V}_\mathrm{C}=\mathop{\sum }\limits_{i=1}^{i=n}{\mathrm{SA}}_{i}\left(\frac{{Z}_{\max }}{{q}_{i}+1}\right)$$

Represents the combined total volume of lakes.

Volume below specific depth (*V*_C*,Z*_):

12 $${V}_{\mathrm{C},z}=\mathop{\sum}\limits_{i=1}^{i=n}{\mathrm{SA}}_{i}\left(\frac{\left({Z}_{\max ,i}-Z\right)\,{\left(1-\frac{Z}{{Z}_{\max ,i}}\right)}^{{q}_{i}}}{{q}_{i}+1}\right)$$

Represents the combined total volume of water below depth *Z*.

Differential volume at depth (d*V*_C*,z*_):

13 $${{\mathrm{d}V}}_{{\rm{C}},z}=\mathop{\sum}\limits_{i=1}^{i=n}{{\mathrm{d}V}}_{z,i}$$

Represents the combined incremental change in lake volume between two consecutive depths (*Z* and *Z* + 1*)*, that is, the sum of the volumes of a depth-specific layer.

#### Physical characteristics of composite lakes

Because larger lakes’ temperature should have more influence on the composite lake temperature profile, we used volume-weighted mean temperature. Each composite lake volume-weighted water temperature profile was determined as the sum of all lakes (*i*) product of each depth (z) mean annual temperature (°C) and differential volume (equation (13)), divided by the sum of all lakes (*i*) differential volumes at each depth:

14 $${T}_{\mathrm{meanC},z}=\frac{{\sum }_{i=1}^{i=n}{T}_{\mathrm{meanZ},i}\times {{\mathrm{d}V}}_{z,i}}{{\sum }_{i=1}^{i=n}{{\mathrm{d}V}}_{z,i}}$$

On the same principle, to calculate the vertically resolved mean temperature of any composite lake, we used the differential volume (equation (13)) as weight factor. Therefore, the composite lake volume-weighted vertically resolved mean water temperature is described as:

15 $${\mathrm{wT}}_{\mathrm{meanC}}=\frac{\sum {T}_{\mathrm{meanC},z}\times {{\mathrm{d}V}}_{{\rm{C}},{\rm{z}}}}{{V}_\mathrm{C}}$$

The composite lake differential area (${{\mathrm{d}A}}_{\mathrm{Iso}C,Z}$) and water volume (${{\mathrm{d}V}}_{\mathrm{Iso}C,Z}$) at depth that is isolated from atmosphere (that is, under thermocline or under ice cover) is calculated from the fraction of the time over one year that any depth is isolated from the atmosphere (fIso_atmZ_) as:

16 $${{\mathrm{d}A}}_{\mathrm{Iso}C,z}=\mathop{\sum}\limits_{i=1}^{i=n}{\mathrm{fIso}}_{\mathrm{atmZ},i}\times {{\mathrm{d}A}}_{z,i}$$

17 $${{\mathrm{d}V}}_{\mathrm{Iso}C,z}=\mathop{\sum}\limits_{i=1}^{i=n}{\mathrm{fIso}}_{\mathrm{atmZ},i}\times {{\mathrm{d}V}}_{z,i}$$

From equation (16) we can calculate the proportion of epilimnetic sediment surface area (in contact with atmosphere via the mixed layer), also known as the fraction of epilimnetic sediment surface area:

18 $${\mathrm{fA}}_{\mathrm{EpiC}}=1-\frac{\sum {{\mathrm{d}A}}_{\mathrm{IsoC},z}}{{\mathrm{SA}}_\mathrm{C}}$$

Similarly, from equation (17), we can estimate the proportion of volume in contact with atmosphere (or fraction of epilimnetic volume; fV_EpiC_) as:

19 $${\mathrm{f}}\,{\mathrm{V}}_{\mathrm{EpiC}}=1-\frac{\sum{{\mathrm{d}V}}_{\mathrm{IsoC},z}}{{V}_\mathrm{C}}$$

Then the ratio of composite epilimnetic volume to sediment surface area (*V*_EpiC _/ *A*_EpiC_) corresponding to the mean thickness of the epilimnion of the composite lake was calculated as:

20 $$\frac{{V}_{\mathrm{EpiC}}}{{A}_{\mathrm{EpiC}}}=\frac{(V_\mathrm{C}-\sum \mathrm{d}{V}_{\mathrm{IsoC,z}})}{\left({\mathrm{SA}}_\mathrm{C}-\right.\sum \left({{\mathrm{d}A}}_{\mathrm{IsoC,z}}\right)}$$

The composite lake mean depth (m) was calculated as the ratio of composite volume to area ratio as:

21 $${Z}_{\mathrm{mean},\mathrm{C}}=\frac{{V}_\mathrm{C}}{{\mathrm{SA}}_\mathrm{C}}$$

The composite lake maximum depth (${Z}_{\max ,\mathrm{C}}$) equals the depth of the deepest lake used in aggregation and the composite lake dynamic ratio^59^ was calculated as:

22 $${\mathrm{DR}}_\mathrm{C}=\frac{\sqrt{{\mathrm{SA}}_\mathrm{C}}}{{Z}_{\mathrm{meanC}}}$$

Where SA_C_ is in km^2^.

#### Process rate scaling

To convert the volumetric process rates (rate_VepiC_) in the water column on an area basis in the epilimnion of the composite lake (rate_AepiC_) we used:

23 $${\mathrm{rate}}_{{A}_{\mathrm{epiC}}}={\mathrm{rate}}_{{V}_{\mathrm{epiC}}}\times \left(\frac{{V}_{\mathrm{epiC}}}{{A}_{\mathrm{epiC}}}\right)$$

To express it per composite lake surface area (rate_AepiC SA_), we used:

24 $${\mathrm{rate}}_{{A}_{\mathrm{epiC}\; \mathrm{SA}}}={\mathrm{rate}}_{{V}_{\mathrm{epiC}}}\times \left(\frac{{V}_{\mathrm{epiC}}}{{A}_{\mathrm{epiC}}}\right)\times {\mathrm{fA}}_{\mathrm{EpiC}}$$

### The 1-by-1 degree grid composite lakes and cluster analysis

In this study, we aggregated depth-specific areas derived from individual lake hypsographic models into composite lake hypsographies at a 1 × 1 degree grid scale, a spatial resolution commonly used in Earth system models. In addition, we performed cluster analysis on these composite lakes to identify patterns and trends. This combined approach of aggregation and clustering bridges local and global analyses, thus enriching our understanding and applicability of data in large-scale environmental modelling.

We calculated 1 × 1 degree grid composite lakes (*N* = 14,477) and carried out hierarchical agglomerative clustering analysis. The cluster analysis was based on three selected features such as: *Z*_meanC_, DR_C_ and fA_epiC_. We selected these composite lakes features because they describe the hypsographic shape well and are not correlated with each other, as most other variables in Table 1 are. Clustering analysis was performed using hierarchical agglomerative clustering with Ward’s method (ward.D2) as implemented in the stats package^60^. In ward.D2, pairwise distances are squared at each merging step to minimize the total variance within each cluster, resulting in groupings that are as internally homogeneous as possible and well separated from one another. Before clustering, the data were standardized using the *scale* function of the same package to ensure uniform contribution of variables to the distance computations, regardless of their original scales. The *hclust* function then iteratively merged clusters according to this variance-minimization criterion. The optimal number of clusters was identified using the silhouette coefficient, calculated with the *silhouette* function of the cluster package^61^. This metric assesses cluster cohesion and separation, with the highest average silhouette score indicating the configuration with the most distinct and well-separated clusters. Such methodological precision ensures the delineation of clusters that are internally homogeneous and externally well differentiated, thus providing robust and clearly defined groupings within the multivariate dataset.

### Propagating uncertainty from individual to composite lakes

Whereas the Imboden model (equation (1)) is simple and highly flexible, it has not yet undergone extensive validation. To this end, we conducted a comprehensive evaluation to assess how well the general hypsography model predicted area at depth (*A*_*Z*_, equation (1)) and volume below depth (*V*_*Z*_, equation (5)) simply from the knowledge of *Z*_max_ and *Z*_mean_ (and by extension *q*; equation (3)) describe the individual lakes for which detailed bathymetric data were available and also test how the model errors propagate at the composite lake level. Therefore, to assess the uncertainty of the modelled *A*_*Z*_ and *V*_*Z*_, we compiled detailed measured bathymetric data (that is, depth maps) and extracted hypsographic curves from a total 3,779 lakes comprising a wide variety of lake morphometries in two different regions, Minnesota and Finland, respectively (Supplementary Table 1).

Using the measured *Z*_max_ and the calculated shape parameter (*q*) from equation (3), we derived the modelled *A*_*Z*_ (equation (1)) and *V*_*Z*_ (equation (5)) at depth intervals of 0.5 m. These predictions, along with measured data, were used to assess the performance of the Imboden model at each depth. We aggregated all *A*_*Z*_ and *V*_*Z*_ and examined the relationship between modelled and measured data to highlight the performance of the model for individual lakes and composite lakes. To evaluate the performance of the hypsography model through depth, we also examined the relationship between the measured *A*_*Z*_ and *V*_*Z*_ vs the relative error (measured − modelled / measured) for both lakes and composite lakes.

We performed an uncertainty analysis to assess the effect of lake morphometry on composite lakes morphometric and physical characteristics, as lake morphometry has been shown to be one of the largest sources of error in biogeochemical model simulations^62^. We analysed the effect of possible variability in composite lakes hypsography arising from potential shortcomings in our modelling approach using predicted parameters *Z*_max_ and *q*. We examine how uncertainties in predicting *Z*_max_ and *q* propagate at the composite lakes level globally and regionally.

We also carried out Monte Carlo simulations to provide a robust framework for evaluating the impact of these uncertainties on our results. Further details are available in Supplementary Methods and Supplementary Text.

### Global model simulations and statistical analyses

The machine learning model development, validation and prediction and the linear regression models were carried out in JMP Pro, Version 16, SAS Institute. All global model simulations were carried out at the High Performance Computing Centre North supercomputing facility, part of the Swedish National Infrastructure for Computing. The lake thermal stratification calculations and all composite lake aggregation algorithms were run using statistical software R^60^ and C^63^.

## Supplementary information

Supplementary Information Supplementary Methods, Text, Figs. 1–9, Tables 1–7 and References.

## Data Availability

The datasets generated and/or analysed, including the data for Figs. 1–6 and Table 1, are available via Zenodo at 10.5281/zenodo.12683317 (ref. ^64^). The data supporting the findings of this study are available from the sources described in Supplementary Table 2. Restrictions may apply to the availability of some of these data, which were used under license for the current study, were not publicly available and must be requested from the data provider.

## Code availability

The relevant code is available via Zenodo at 10.5281/zenodo.12683317 (ref. ^64^). This includes README files and flowcharts that provide an overview of each script’s specific role in data processing, analysis or visualization.

## References

[1] Timms, B. V. *Lake Geomorphology* (Gleneagles Publishing, 1992).

[2] Lehner, B. & Döll, P. Development and validation of a global database of lakes, reservoirs and wetlands. *J. Hydrol.* **296**, 1–22 (2004).

[3] Pekel, J. F., Cottam, A., Gorelick, N. & Belward, A. S. High-resolution mapping of global surface water and its long-term changes. *Nature* **540**, 418–422 (2016). 27926733 10.1038/nature20584

[4] Amante, C. & Eakins, B. W. *ETOPO1 1 Arc-minute Global Relief Model: Procedures, Data Sources and Analysis* (NOAA, 2009).

[5] Eakins, B. W. & Sharman, G. F. *Hypsographic Curve of Earth’s Surface from ETOPO1* (NOAA National Geophysical Data Center, 2012).

[6] Carpenter, S. R. Lake geometry: implications for production and sediment accretion rates. *J. Theor. Biol.* **105**, 273–286 (1983).

[7] Ferland, M.-E., Prairie, Y. T., Teodoru, C. & del Giorgio, P. A. Linking organic carbon sedimentation, burial efficiency, and long-term accumulation in boreal lakes. *J. Geophys. Res. Biogeosci.* **119**, 836–847 (2014).

[8] Vadeboncoeur, Y., Peterson, G., Vander Zanden, M. J. & Kalff, J. Benthic algal production across lake size gradients: interactions among morphometry, nutrients, and light. *Ecology* **89**, 2542–2552 (2008). 18831175 10.1890/07-1058.1

[9] Magee, M. R. & Wu, C. H. Response of water temperatures and stratification to changing climate in three lakes with different morphometry. *Hydrol. Earth Syst. Sci.* **21**, 6253–6274 (2017).

[10] Fee, E. J. A relation between lake morphometry and primary productivity and its use in interpreting whole-lake eutrophication experiments. *Limnol. Oceanogr.* **24**, 401–416 (1979).

[11] Livingstone, D. M. & Imboden, D. M. The prediction of hypolimnetic oxygen profiles: a plea for a deductive approach. *Can. J. Fish. Aquat. Sci.* **53**, 924–932 (1996).

[12] Bastviken, D., Cole, J. J., Pace, M. L. & Van de Bogert, M. C. Fates of methane from different lake habitats: connecting whole‐lake budgets and CH_4_ emissions. *J. Geophys. Res. Biogeosci*. **113**, G02024 (2008).

[13] Vadeboncoeur, Y. in *Encyclopedia of Inland Waters* (ed Likens G. E.) 52–59 (Academic Press, 2009).

[14] Woolway, R. I. et al. Phenological shifts in lake stratification under climate change. *Nat. Commun.* **12**, 2318 (2021). 33875656 10.1038/s41467-021-22657-4 PMC8055693

[15] Downing, J. A. et al. The global abundance and size distribution of lakes, ponds, and impoundments. *Limnol. Oceanogr.* **51**, 2388–2397 (2006).

[16] Seekell, D. A., Pace, M. L., Tranvik, L. J. & Verpoorter, C. A fractal-based approach to lake size-distributions. *Geophys. Res. Lett.* **40**, 517–521 (2013).

[17] Cael, B. B., Biggs, J. & Seekell, D. A. The size-distribution of earth's lakes and ponds: limits to power-law behavior. *Front. Environ. Sci.* **10**, 888735 (2022).

[18] Cael, B. B. & Seekell, D. A theory for the relationship between lake surface area and maximum depth. *Limnol. Oceanogr. Lett.* **7**, 527–533 (2022).

[19] Soranno, P. A. et al. Spatial and temporal variation of ecosystem properties at macroscales. *Ecol. Lett.* **22**, 1587–1598 (2019). 31347258 10.1111/ele.13346

[20] MacKay, M. D. et al. Modeling lakes and reservoirs in the climate system. *Limnol. Oceanogr.* **54**, 2315–2329 (2009).

[21] DelSontro, T., Beaulieu, J. J. & Downing, J. A. Greenhouse gas emissions from lakes and impoundments: upscaling in the face of global change. *Limnol. Oceanogr. Lett.* **3**, 64–75 (2018). 10.1002/lol2.10073 PMC7029703 32076654

[22] Levin, S. A. The problem of pattern and scale in ecology. *Ecology* **73**, 1943–1967 (1992).

[23] Soranno, P. A. et al. Cross‐scale interactions: quantifying multi‐scaled cause–effect relationships in macrosystems. *Front. Ecol. Environ.* **12**, 65–73 (2014).

[24] Cael, B. B., Heathcote, A. J. & Seekell, D. A. The volume and mean depth of Earth’s lakes. *Geophys. Res. Lett.* **44**, 209–218 (2017).

[25] Hollister, J. W., Milstead, W. B. & Urrutia, M. A. Predicting maximum lake depth from surrounding topography. *PLoS ONE* **6**, e25764 (2011). 21984945 10.1371/journal.pone.0025764 PMC3184154

[26] Minns, C. K., Moore, J. E., Shuter, B. J. & Mandrak, N. E. A preliminary national analysis of some key characteristics of Canadian lakes. *Can. J. Fish. Aquat. Sci.* **65**, 1763–1778 (2008).

[27] Heathcote, A. J., del Giorgio, P. A., Prairie, Y. T. & Brickman, D. Predicting bathymetric features of lakes from the topography of their surrounding landscape. *Can. J. Fish. Aquat. Sci.* **72**, 643–650 (2015).

[28] Sobek, S. Predicting the depth and volume of lakes from map-derived parameters. *Inland Waters* **1**, 177–184 (2011).

[29] Oliver, S. K. et al. Prediction of lake depth across a 17-state region in the United States. *Inland Waters* **6**, 314–324 (2016).

[30] Woolway, R. I. et al. Global lake responses to climate change. *Nat. Rev. Earth. Env.* **1**, 388–403 (2020).

[31] Kelly, C. A. et al. Natural variability of carbon dioxide and net epilimnetic production in the surface waters of boreal lakes of different sizes. *Limnol. Oceanogr.* **46**, 1054–1064 (2001).

[32] Pace, M. L. & Prairie, Y. T. in *Respiration in Aquatic Ecosystems* (eds del Giorgio P. A. & Williams P. J. L. B.) 103–121 (Oxford Univ. Press, 2005).

[33] Seekell, D., Cael, B., Norman, S. & Byström, P. Patterns and Variation of Littoral Habitat Size Among Lakes. *Geophys. Res. Lett.* **48**, e2021GL095046 (2021).

[34] Einsele, G. & Hinderer, M. Quantifying denudation and sediment–accumulation systems (open and closed lakes): basic concepts and first results. *Palaeogeogr. Palaeoclimatol. Palaeoecol.* **140**, 7–21 (1998).

[35] Zhang, B., Schwartz, F. W. & Liu, G. Systematics in the size structure of prairie pothole lakes through drought and deluge. *Water Resour. Res.* **45**, W04421 (2009).

[36] Meybeck, M. in *Physics and Chemistry of Lakes* Vol. 14 (eds Imboden D. et al.) 1–35 (Springer, 1995).

[37] Clarke, G., Leverington, D., Teller, J. & Dyke, A. Paleoclimate. Superlakes, megafloods, and abrupt climate change. *Science* **301**, 922–923 (2003). 12920285 10.1126/science.1085921

[38] Ehlers, J., Gibbard, P. L. & Hughes, P. D. (eds) in *Quaternary Glaciations - Extent and Chronology* Vol. 15 1–14 (Elsevier, 2011).

[39] Beck, H. E. et al. Present and future Koppen-Geiger climate classification maps at 1-km resolution. *Sci. Data* **5**, 180214 (2018). 30375988 10.1038/sdata.2018.214 PMC6207062

[40] Jane, S. F. et al. Widespread deoxygenation of temperate lakes. *Nature* **594**, 66–70 (2021). 34079137 10.1038/s41586-021-03550-y

[41] Zhao, M. et al. The GFDL Global Atmosphere and Land Model AM4.0/LM4.0: 1. Simulation characteristics with prescribed SSTs. *J. Adv. Model. Earth Syst.* **10**, 691–734 (2018).

[42] Rockström, J. et al. A safe operating space for humanity. *Nature* **461**, 472–475 (2009). 19779433 10.1038/461472a

[43] Richardson, K. et al. Earth beyond six of nine planetary boundaries. *Sci. Adv.* **9**, eadh2458 (2023). 37703365 10.1126/sciadv.adh2458 PMC10499318

[44] Steffen, W. et al. Sustainability. Planetary boundaries: guiding human development on a changing planet. *Science* **347**, 1259855 (2015). 25592418 10.1126/science.1259855

[45] Carpenter, S. R. & Bennett, E. M. Reconsideration of the planetary boundary for phosphorus. *Environ. Res. Lett.* **6**, 014009 (2011).

[46] Müller, B., Bryant, L. D., Matzinger, A. & Wüest, A. Hypolimnetic oxygen depletion in eutrophic lakes. *Environ. Sci. Technol.* **46**, 9964–9971 (2012). 22871037 10.1021/es301422r

[47] Zwart, J. A. et al. Cross‐scale interactions dictate regional lake carbon flux and productivity response to future climate. *Geophys. Res. Lett.* **46**, 8840–8851 (2019).

[48] Meybeck, M., Green, P. & Vorosmarty, C. A new typology for mountains and other relief classes: an application to global continental water resources and population distribution. *Mt. Res. Dev.* **21**, 34–45 (2001).

[49] Global landform classification (GIS raster dataset) (2020). *ESDAC* https://esdac.jrc.ec.europa.eu/content/global-landform-classification (2020).

[50] Imboden, D. M. Limnologische transport-und näihrstoffmodelle. *Schweiz. Z. Hydrol.* **35**, 29–68 (1973).

[51] Amatulli, G. et al. A suite of global, cross-scale topographic variables for environmental and biodiversity modeling. *Sci. Data* **5**, 180040 (2018). 29557978 10.1038/sdata.2018.40 PMC5859920

[52] QGIS Development Team. QGIS geographic information system. *Open Source Geospatial Foundation Project* http://qgis.org (2020).

[53] Goudsmit, G. H., Burchard, H., Peeters, F. & Wüest, A. Application of k‐ϵ turbulence models to enclosed basins: the role of internal seiches. *J. Geophys. Res. Oceans* **107**, 3230–3242 (2002).

[54] Kobler, U. G. & Schmid, M. Ensemble modelling of ice cover for a reservoir affected by pumped‐storage operation and climate change. *Hydrol. Process.* **33**, 2676–2690 (2019).

[55] Mesman, J. P. et al. Performance of one-dimensional hydrodynamic lake models during short-term extreme weather events. *Environ. Modell. Software* **133**, 104852 (2020).

[56] Thiery, W. et al. LakeMIP Kivu: evaluating the representation of a large, deep tropical lake by a set of one-dimensional lake models. *Tellus A: Dyn. Meteorol. Oceanogr.* **66**, 21390 (2014).

[57] Golub, M. et al. A framework for ensemble modelling of climate change impacts on lakes worldwide: the ISIMIP lake sector. *Geosci. Model Dev.* **15**, 4597–4623 (2022).

[58] Wilson, H. L. et al. Variability in epilimnion depth estimations in lakes. *Hydrol. Earth Syst. Sci.* **24**, 5559–5577 (2020).

[59] Håkanson, L. Lake bottom dynamics and morphometry: the dynamic ratio. *Water Resour. Res.* **18**, 1444–1450 (1982).

[60] R Core Team. R: a language and environment for statistical computing (R Foundation for Statistical Computing, 2020).

[61] Maechler, M., Rousseeuw, P., Struyf, A., Hubert, M. & Hornik, K. cluster: Cluster analysis basics and extensions. R package version 2.1.8.1 (CRAN, 2025); https://CRAN.R-project.org/package=cluster

[62] Zwart, J. A. et al. Spatially explicit, regional‐scale simulation of lake carbon fluxes. *Glob. Biogeochem. Cycles* **32**, 1276–1293 (2018).

[63] Stroustrup, B. *The C++ Programming Language* 2nd edn (Addison-Wesley, 1995).

[64] Gudasz, C., Prairie, Y. T. & Vachon, D. A comprehensive framework for integrating lake hypsography and function on a global scale. *Zenodo*10.5281/zenodo.12683317 (2025). 10.1038/s44221-025-00461-4 PMC12279543 40704046

