# Supplementary material for: A comprehensive framework for integrating lake hypsography and function on a global scale
Source: Nat Water. 2025 Jul 17;3(7):818–30. doi: 10.1038/s44221-025-00461-4 (PMC12279543; doi:10.1038/s44221-025-00461-4)
Supplement: Supplementary file 1 — Supplementary Methods, Text, Figs. 1–9, Tables 1–7 and References. [file 44221_2025_461_MOESM1_ESM.pdf]

nature water

Supplementary information https://doi.org/10.1038/s44221-025-00461-4

A comprehensive framework for integrating lake hypsography and function on a global scale

In the format provided by the authors and unedited

Supplementary Information

Table of Contents

Supplementary Methods, 2

Global lake map, 2
Lake bathymetry data, 2
Physical dynamics, 2
Propagating uncertainty from individual to composite lakes, 3

Supplementary Text - Propagating uncertainty, 4

Supplementary Figures,

Fig. S1 Graphical idealization of the general hypsography model, 6
Fig. S2 Flowchart of the methods and analyses used in creating composite lakes, 7
Fig. S3 Lake morphometry dataset used for model development and validation, 8
Fig. S4 Random forests models of Z_{mean}, Z_{max} and q for individual lake predictions, 9
Fig. S5 The measured and modeled areas at depth of lakes in Minnesota and Finland, 10
Fig. S6 The measured and modeled volumes below depth of lakes in Minnesota and Finland, 11
Fig. S7 The relationship between the number of lakes in the composite and relative error, 12
Fig. S8 Boxplots illustrating the predictred and resampled hypsography of individual lakes, 12
Fig. S9 Composite lakes variability across global and climate regions, 13

Supplementary Tables,

Table S1 Summary of lake characteristics of lakes with measured bathymetry, 14
Table S2 The data sources and availability, 14
Table S3 Summary of the random forest models goodness of fit for the prediction of Z_{mean}, Z_{max} and q, 16
Table S4 The one-degree composite lake clusters and their morphometry parameters, 16
Table S5 Results of the relative measured and modeled linear models, 16
Table S6 Results of the absolute measured and modeled linear models, 17
Table S7 The global and regional composite lake morphometry parameters, 17

References, 17

Supplementary Methods

Global lake map

The increasing availability of satellite imagery resulted in several studies developing maps representing rasters of global freshwaters[1-3]. However, there is no comprehensive evaluation of these global maps. We used the seasonally permanent freshwater surface cover rasters of global surface water (GSW)[1] to derive a global lake map. We chose the permanent surface waters during 2018 in the seasonality raster of GSW as the basis for our natural lakes map (GSWL). The GSW dataset does not discriminate between natural lakes from streams, rivers, and man-made reservoirs. Hence, we used the vector maps of the global extent of streams and river widths that generally describe large systems[4]. Due to the 30m resolution of the satellite imagery in the GSW raster, it was observed that smaller water bodies, specifically streams and small rivers, are generally not captured. However, we acknowledge that small fragments representing streams are occasionally present in the dataset, but this does not constitute a systematic issue. To mitigate errors, especially in shoreline delineation of smaller lakes, we deliberately set a lower size limit of 1ha for our lake mapping. In addition, we used the Global Reservoir and Dam database (with the exception of Lake Baikal, Ontario, Onega and Victoria, which were considered natural)[5] to mask the GSW raster. Following the GSW masking we converted the raster to vector. We limited lake size coverage to lakes larger than 1ha. We used Google's Earth Engine for the GSW masking operations[6] and QGIS[7] for raster to vector conversions. Furthermore, to fix the vector errors resulting from the raster conversion we used PostGIS[8]. The map is based on geographic coordinate system WGS84 and geodesic areas were calculated for the resulting lake polygons. The resulting global map of natural lakes (GSWL) was used subsequently in our analyses and was limited to lakes > 1ha. One of the better developed and widely used lake map, HydroLAKES[9], includes lakes >10ha, which would have meant excluding from our analysis the 1-10 ha lake size category, comprising approximately 4.1 million lakes (GSWL). While acknowledging that the GSW dataset is not devoid of errors, it's important to note that these inaccuracies are not systematic and do not substantially impact the overall findings of our study.

Lake bathymetry data

Using available bathymetric points, we generated hypsographic curves for 1871 lakes in Finland[10] and 1908 lakes in Minnesota[11]. We used Tinfour 2.1.7[12], fast an accurate algorithm, to triangulate bathymetric points and derive lake hypsography curves at 0.5m intervals, including calculations of lake surface areas, volumes, Z_{mean}, Z_{max}, A_Z, and V_Z. For lakes in Minnesota, we used the available bathymetric raster data at 5m pixel resolution as the basis to derive bathymetric points.

Physical dynamics

We used the ERA5-Land climate reanalysis data from the European Centre for Medium-Range Weather Forecasts, 0.1°/0.1° grids, 9 km native resolution for the period 2017-2018 as forcings to drive the physical lake model[13]. Daily mean data, aggregated from 1-hourly ERA5 data, were downloaded in monthly files from the Copernicus Climate Change Service (C3S), accessed on 2022-03-06 and 2022-03-07. The model forcing was applied daily, at a 300 second simulation time step, using interpolated daily mean values and a vertical resolution of 0.1 m for all lakes. Specifically, the climate variables required for physical model simulations were surface solar radiation downwards, surface pressure, the 10 m wind velocity components, v (meridional; north–south) and u (zonal; west–east), 2 m temperature and 2 m dewpoint temperature (i.e., Simstrat forcing mode 2). The climate data was then extracted at the centroid

coordinate for each of the 5.74 million lake polygons in our dataset and subsequently used to create the forcing data file. We used the wind sheltering formulation of Hondzo and Stefan[14] based on the surface area of each system. Water transparency, as described by the vertical attenuation coefficient of light (k_d, m^{-1}) is an important parameter governing lake physics and overall energy balance. However, reliable global lake k_d values are currently lacking. Previous studies have used a constant and global value of $k_d = 3\ m^{-1}$ [15]. This would be similar to very low water transparency value as Secchi depth of approximately 0.6 m. Others have used lower constant values with $k_d = 1\ m^{-1}$ [16]. For this study, we used a low and constant k_d value of 0.57 m^{-1} that characterizes a low, base level of water transparency for the simulation period. This was calculated based on the relationship with Secchi depth (Z_{SD}) using the equation $k_d = 1.7/Z_{SD}$ [17], and a median Z_{SD} value of 3 m representing epilimnetic water quality of 14657 lakes[18]. Other studies[19] attempt to use a lake specific transparency based on lake depth[20]. However, such estimates are highly uncertain for individual lake level predictions. The model was initialized with a constant temperature of 4°C for all depths. The model simulations were conducted over a period of two years, namely 2017-2018. However, only the simulation data from the year 2018 was utilized for subsequent analyses, while the data from 2017 was used to stabilize the model simulations. We used the output files containing temperature, vertical diffusivity, and ice data for further analyses.

The physical dynamic simulations carried out using Simstrat, a physical modeling platform well-tested across diverse lake morphometries and climate[21-24], is well-suited to explore lake dynamics broadly. Our simulations explicitly incorporated the predicted hypsography of all individual lakes larger than 1 ha, in contrast to simplified geometric assumptions[19,24-26]. Because of the large computing resources required to perform our global analysis (≈1 million core-h, and 30 TB of compressed storage), we limited our simulations to the use of a single model (Simstrat). Future steps should involve a comparison with model ensemble to mitigate individual model weaknesses[26,27], an exercise that was beyond the scope of our main objective of describing the conceptual framework presented here.

Propagating uncertainty from individual to composite lakes

The global lake dataset, comprising 5735587 lakes, inherently contains variability and uncertainties in predicting Z_{max} and q needed for hypsography modeling. In developing the predictive models for Z_{max} and q, we observed a non-normal distribution of prediction errors. We applied a cube root transformation to address this issue, which helped stabilize the variance and approximate normality in the errors. The uncertainties in our modeling were quantified using the RMSE values derived from the model validation, as outlined in Supplementary Table S3. For Z_{max}, lakes with an area of 10 km^2 or greater were assigned a value of 0.738, while for those smaller, we used a value of 0.450. Similarly, for the parameter q, the assigned values were 0.450 for lakes 10 km^2 or larger and 0.350 for those smaller. Subsequently, we used this cube root transformed error structure to perform 100 iterations of Monte Carlo simulations for all lakes with predicted Z_{max} and q, randomly resampling from a truncated normal distribution. The 39,808 lake morphometry data informed the truncation thresholds to ensure that the resampled values remained within plausible physical boundaries. Therefore, we set Z_{max} between 1 and 1000 m and 0.2 and 82 for q. Notably, 115 lakes larger than 400 km^2 where data were available retained their original Z_{max} and q values without resampling.

We used Gelman-Rubin diagnostics and Effective Sample Sizes (ESS) from the *Coda* R package[28] to validate resampling. The convergence diagnostics highlighted the reliability of our resampling simulation. The Gelman-Rubin diagnostic pointed to potential scale reduction factors of 1 for Z_{max} and q, suggesting a robust convergence of our Markov chains. Furthermore, ESS showcased efficient parameter space exploration and minimal autocorrelation, and 99.98 and 99.99 % of resampled Z_{max} and q, respectively, were independent. The multivariate

potential scale reduction factor, at a value of 1, further corroborated these findings, indicating the convergence of chains to a uniform joint distribution.

The resampled values of Z_{max} and q were used to model the hypsography of individual lakes, subsequently aggregating them to create global and climate region composite lakes for each of the 100 iterations of the Monte Carlo simulation. We calculated the average and SD of A_Z and V_Z (Supplementary Table S7). For each of the 100 iterations, we calculated composite lakes features such as Z_{meanC}, Z_{maxC}, V_C, and DR_C and then their average and SD. Since the composite lakes SA_C is fixed, the calculated SD represents a measure of variability in estimating the area from the hypsography curve. By integrating these variations, we were able to compute a consolidated SD that effectively captures the overall variability in area and volume across the hypsographic curves of the composite lakes. The integrated SD for area or volume ($ISD_{A,V}$) was calculated as follows:

$$ISD_{A,V} = \sqrt{\sum_Z \left(SD_{Az,Vz}\right)^2} \qquad (1)$$

Where: $SD_{Az,Vz}$ is the standard deviation of the area or volume at depth Z. The sum is taken over all depths.

To calculate the average and SD of the epilimnetic volume (V_{EpiC}) and sediment surface area (A_{EpiC}) for composite lakes, we relied on the SD of V_Z and A_Z across various depths, derived from 100 iterations of Monte Carlo simulations. We first multiplied the total composite volume and area from each of the 100 iterations of the Monte Carlo simulations, by the epilimnetic volume (fV_{EpiC}) and sediment surface area (fA_{EpiC}) fractions from Tables 1 to derive V_{EpiC} andA_{EpiC}. We then computed the integrated standard deviation (ISD) for total volume and total area (Eq. 23). The SDs of V_{EpiC} and A_{EpiC} were then calculated by multiplying the ISD of the volume and area by the absolute values of fV_{EpiC} and fA_{EpiC}, respectively. Next, we performed a 10000-iteration Monte Carlo simulation, generating random samples from a normal distribution for V_{EpiC} and A_{EpiC} based on their means and respective SDs and then calculated the ratio V_{EpiC}/A_{EpiC} for each sample. The SD of these ratio values provided the uncertainty in V_{EpiC}/A_{EpiC}. The average and SD of morphometry parameters of the global and regional composite lakes, based on resampled morphometry data and Monte Carlo simulations, are described in Supplementary Table S7.

Supplementary Text - Propagating uncertainty

From lakes to composite lakes, we can identify three main categories of error: 1. Errors inherent to the general hypsography model itself (Methods, Eqs. 1 and 5), 2. Errors that arise in measuring and predicting the Z_{max}, Z_{mean} and q, and 3. Mapping errors due to echosounding of lake depths and delimiting surface areas.

We evaluated the suitability of the general hypsography model (Methods, Eq. 1) to describe individual lake hypsography using measured bathymetric data in a combined dataset of 3,779 lakes from Minnesota and Finland (see Supplementary Table S1 and Methods). Our analysis revealed a uniform spread around the 1:1 line of measured vs. modeled area at depth (A_Z) (Supplementary Figure S5a) and a robust fit in both relative (Supplementary Table S5) and absolute terms (Supplementary Table S6). Notably, most relative errors (observed – predicted/observed) of modeled A_Z ranged within ±35% (Supplementary Figure S5c). More substantial relative errors were evident in less than 20% of the predicted areas, highlighting the model's limitations in accurately predicting small relative areas at the greatest depths. Similarly, modeled volumes below depth Z (V_Z, Eq. 5 Methods) exhibited a good fit (Supplementary Figure S6a, Supplementary Tables S5,6) and a similar increase in relative errors for smaller relative volumes at higher depths. The relative errors for modeled V_Z ranged

from -25 to +50% (Supplementary Figure S6c). More importantly, when aggregating areas (Supplementary Figure S5b) and volumes (Supplementary Figure S6b) of all these lakes, the composite lake areas and volumes prediction closely aligned with observed data on the 1:1 line, yielding minor relative errors, 2-3% for area (Supplementary Figure S5d) and 0.1-0.2% for volume (Supplementary Figure S6d). The relative errors for both areas and volumes at depth increase with decreasing numbers of lakes used in aggregation (Supplementary Figure S7). Hence, the general hypsography model used here reasonably described lake hypsography, and the errors associated with modeling individual lake areas and volumes at depth propagate to a small degree when aggregating to the composite lakes.

Predictive models of lake morphometry parameters, such as Z_{mean} and Z_{max}, have been developed in numerous studies[9,29-33]. Regardless of the approach, the individual lake depth predictions from these methods carry a large degree of uncertainty, which can span an order of magnitude. However, aggregating depth data from a larger number of individual lake predictions proved to be closer to the observed values, as in the case of total regional lake volume[33,34]. Aggregated lake hypsography may lead to more accurate representation due to a larger sample size and by reducing noise and random variability. While the models predicting Z_{max} and q developed in this study performed reasonably well, the individual prediction showed substantial uncertainty, especially for lakes <10 km² (Supplementary Table S3). However, these models effectively capture the overall trend of increasing depth with increasing lake size. The purpose of the lake dataset with predicted Z_{max} and q was to function as input parameters for the Imboden model (Equation 1, Methods), which was used to generate individual lake hypsography and then aggregate it at the composite lake level. Consequently, it is imperative to evaluate the impact of the modeled errors in Z_{max} and q on the hypsography of individual lakes and their cumulative effect at the composite lakes level. By employing 100 iterations of the Monte Carlo simulation, we randomly resampled the uncertainties associated with the model validation of Z_{max} and q (see Methods). While Supplementary Figure S8a delineates the variability inherent in modeling hypsography with predicted Z_{max} and q, Supplementary Figure S8b depicts the relative area variability arising from 100 iterations of resampling Z_{max} and q. Specifically, the A_Z interquartile range (25th to 75th percentile) in the boxplots expanded substantially, confirming the increase in variation.

Intriguingly, the global and regional hypsography (Supplementary Figure S9) and corresponding features (Supplementary Table S7) remained remarkably stable when subjected to these randomly resampled Z_{max} and q across 100 iterations. The global composite A_Z relative error (predicted-resampled/predicted) mean and SD was -0.74±1.26%. Similarly, using measured data to model hypsography, the errors canceled out at the composite lake level compared to measured data for composite lake A_Z (Supplementary Figure S5b) and V_Z (Supplementary Figure S6b). In Tropical and Polar regions, the lack of morphometry data for some large lakes meant that resampling Z_{max} and q resulted in a variation increase as reflected in the hypsography curves (Supplementary Figure S9) and features (Supplementary Table S7). The Pareto distribution of lake size abundance is a fundamental property of lake populations. In the context of aggregating lake hypsography, this distribution implies that larger and fewer lakes may exert an outsized influence on the hypsography of the composite lake. Moreover, the uncertainty of hypsography varies according to lake size, with fewer lakes resulting in less cancellation effect and greater weight due to their size and depth. Based on data for 115 of the 240 lakes larger than 400 km^2, the influence of the remaining lakes on the global and regional composite lake hypsography was small with resampled lake Z_{max} and q, as indicated in Supplementary Figures S8,9. At the same time, having hypsographic data of large lakes can stabilize the composite lakes hypsography by the large area and volume contribution against more variable small lakes. Interestingly, the analysis suggests that changes in the Pareto distribution describing lake size distribution will have a more substantial impact on the global and regional hypsography of the composite lakes than the lake hypsography modeling

uncertainty. Therefore, understanding and predicting the changes in lake abundance is crucial for accurately modeling the composite lakes hypsography. In this context, mapping errors of lake areas, of smaller lakes, can add errors. The lake map derived from Landsat imagery at 30 m resolution[1] has limitations for identifying water bodies less than 1ha and delineating lake shorelines. However, for the purpose of the analysis discussed, it is deemed acceptable. Because of the inherent but unknown uncertainty in the measured bathymetric data stemming from the depth sounding measurements and depth interpolation method, our analysis does not incorporate this error propagation to the lake and composite lakes hypsography.

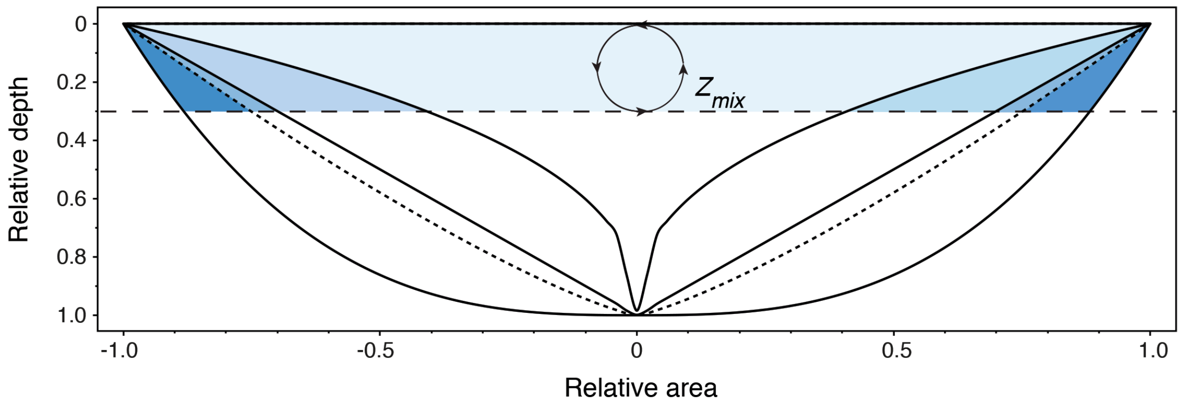

Supplementary Figure S1. Graphical idealization of the general hypsography model. Diagram of the 2D lake shapes describing the change in relative area with relative depth of the hypsography model with *q* parameter of 0.7, 1.6, 2, 5 (solid lines). The dashed line corresponds to the average *q* estimate (q = 1.6) obtained for all lakes. The values of *q* (<2) correspond to cuvette-shaped lake basins, while large values ($q > 4$) are representative of flat lakes with a small central deep area. The q=2 is equivalent to a cone lake basin shape. The short dash line corresponds to the lake shape as a cone. The long dash represents the mixed layer depth. The increasingly darker blue colors mark the decrease in sediment surface area exposed to mixing waters with increasingly cuvette-shaped type of lakes.

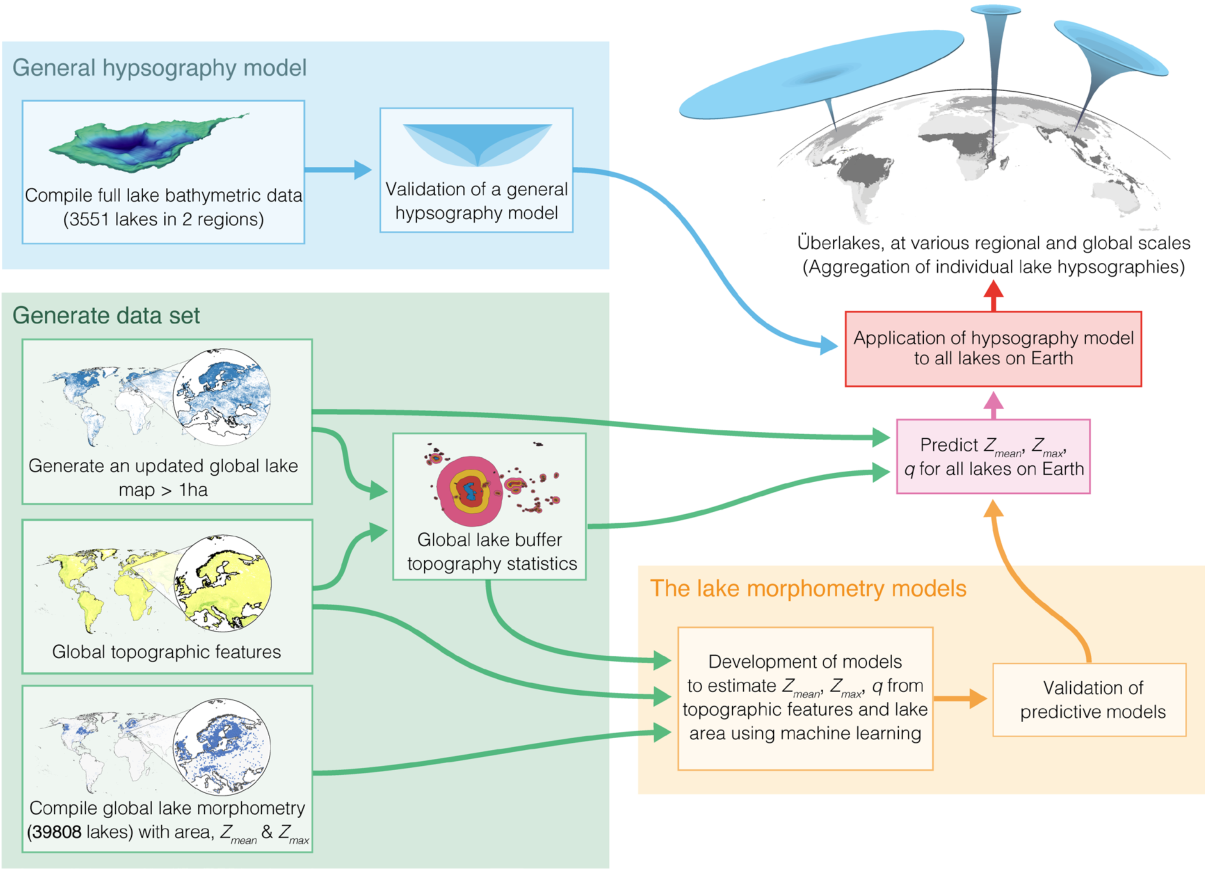

Supplementary Figure S2. Flowchart of the methods and analyses used in creating composite lakes.

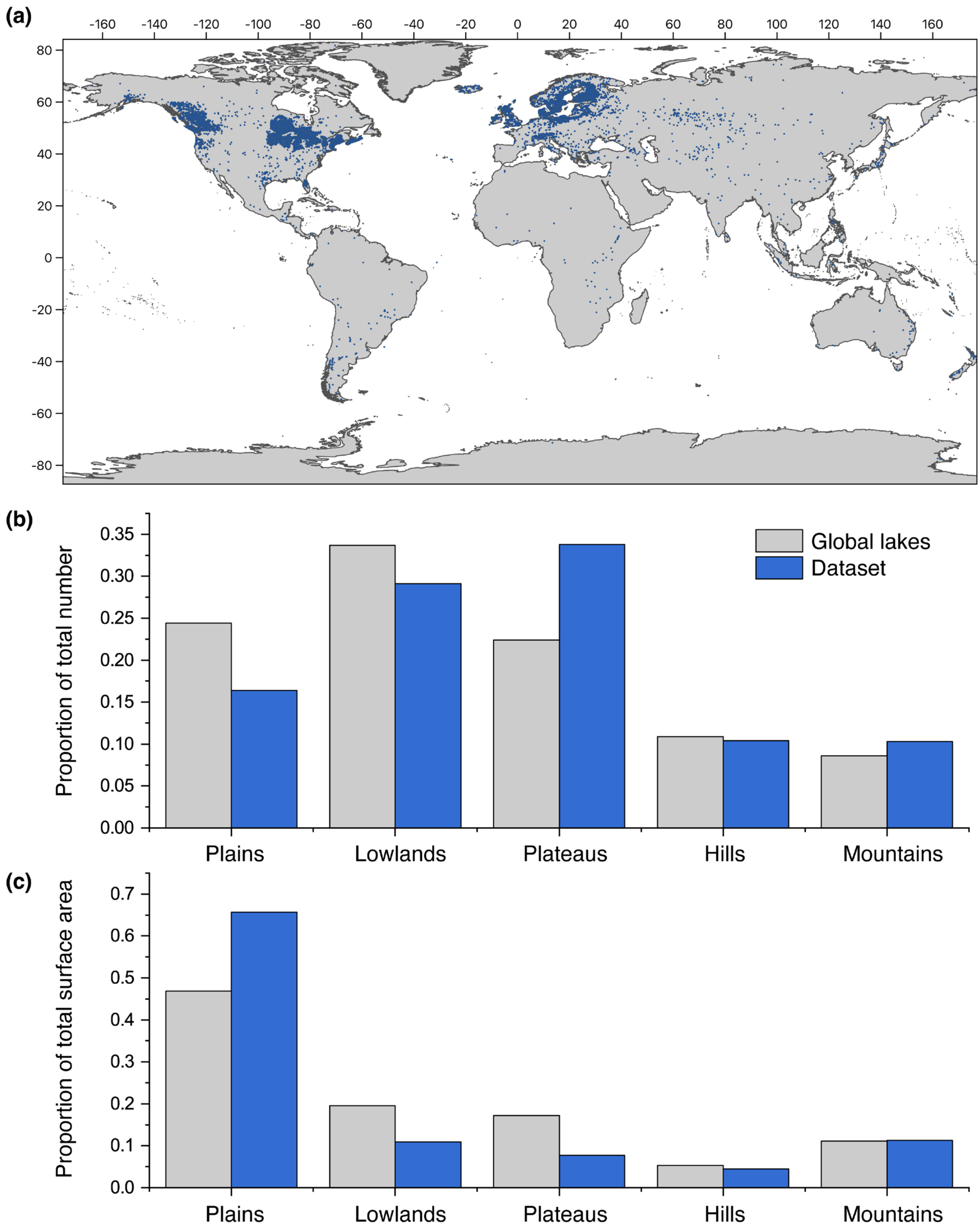

Supplementary Figure S3. Lake morphometry dataset used for model development and validation. (**a**) Global map with the location of the lakes. (**b**) Proportion of the total lake numbers. (**c**) Proportion of total surface area in major landforms. Global landform classification[35], using the 0.5° raster data[36]. Landform classes were aggregated to reflect the five major categories. The data source can be found in Supplementary Table S2.

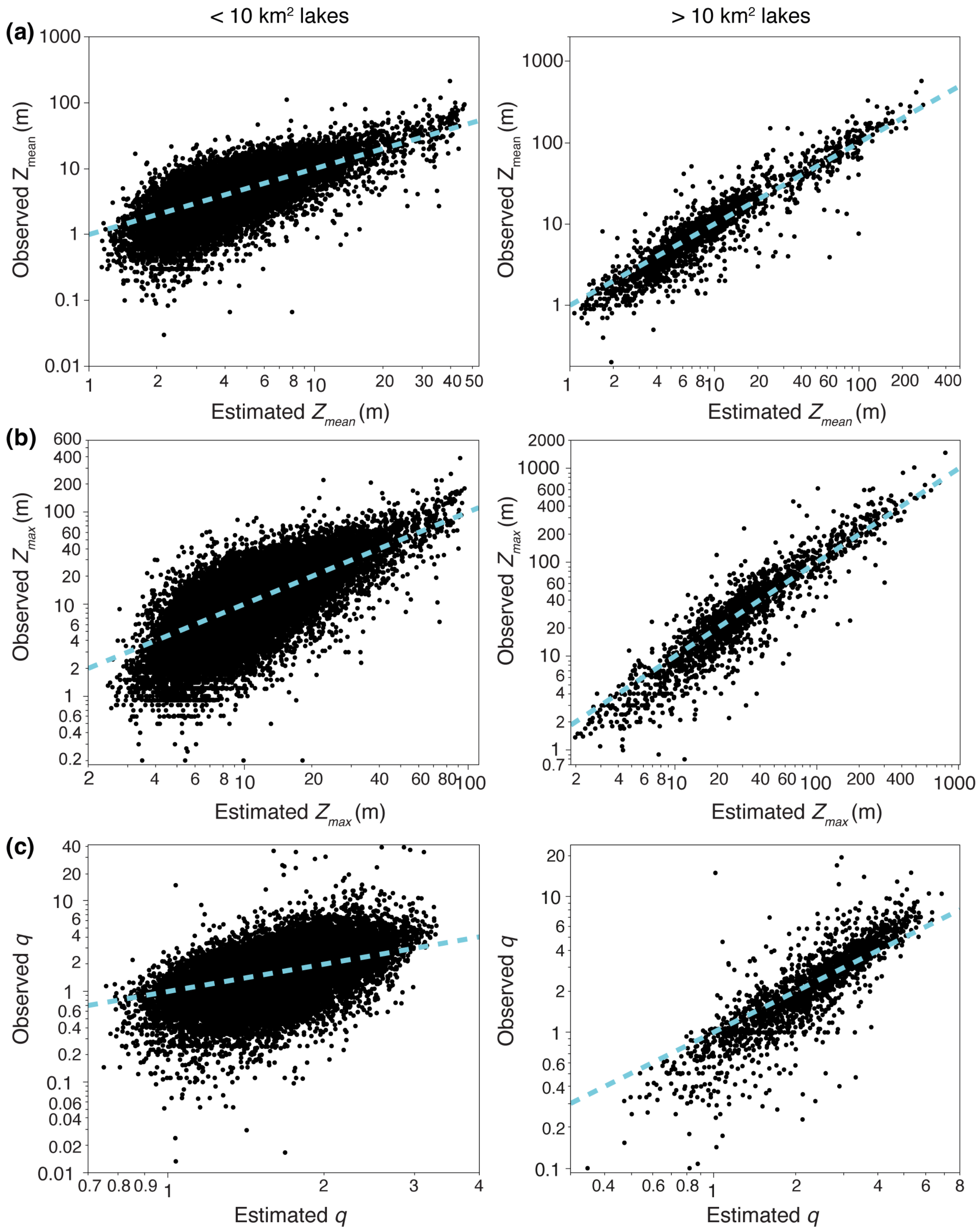

Supplementary Figure S4. Random forests models of Z_{mean}, Z_{max} and q for individual lake predictions. The observed vs. predicted values of the (**a**) Z_{mean}, (**b**) Z_{max}, and (**c**) q parameters developed for small (0.002 < Lake Area < 10km^2) and large (>10km^2) lakes, respectively. The data on both axes is shown on a log10 scale. Dashed lines correspond to the 1:1 line.

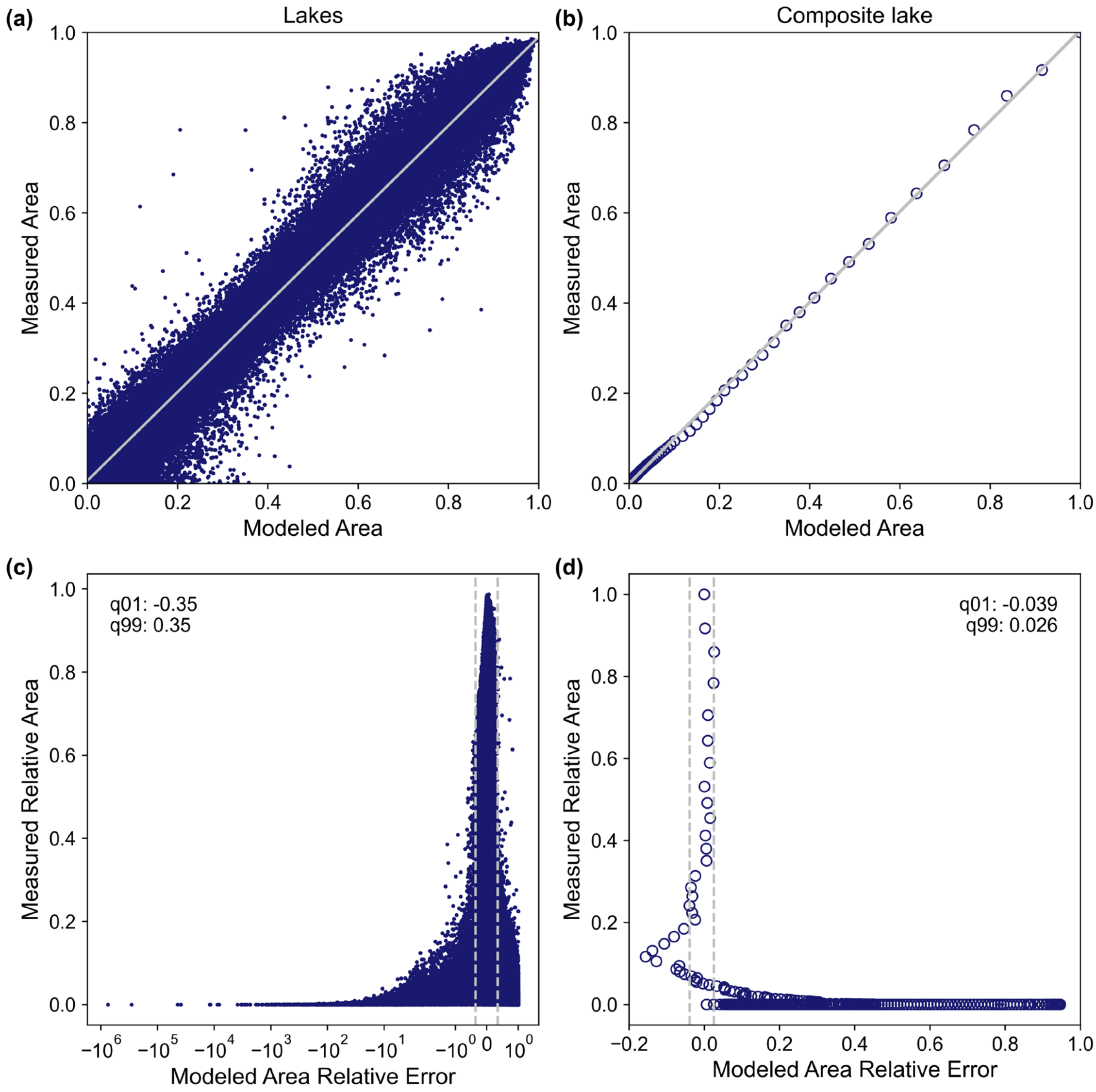

Supplementary Figure S5. The measured and modeled areas at depth of lakes in Minnesota and Finland. (**a**) The relationship between measured and modeled area at depth (Eq. 1, Methods) of individual lakes. (**b**) The aggregated areas at depth (Eq. 9, Methods) of the composite lake. (**c**) The relative errors for the modeled area at depth of the individual lakes (symlog scale) and (**d**) composite lake. The red dashed lines mark the 1st and 99th percentiles of the relative areas at depths exceeding 0.2. The summary of the linear model fit in relative and absolute terms for lakes (**a**) and composite lake (**b**) can be found in Supplementary Tables S5 and S6, respectively. Bathymetry data from Minnesota and Finland (Supplementary Table S1).

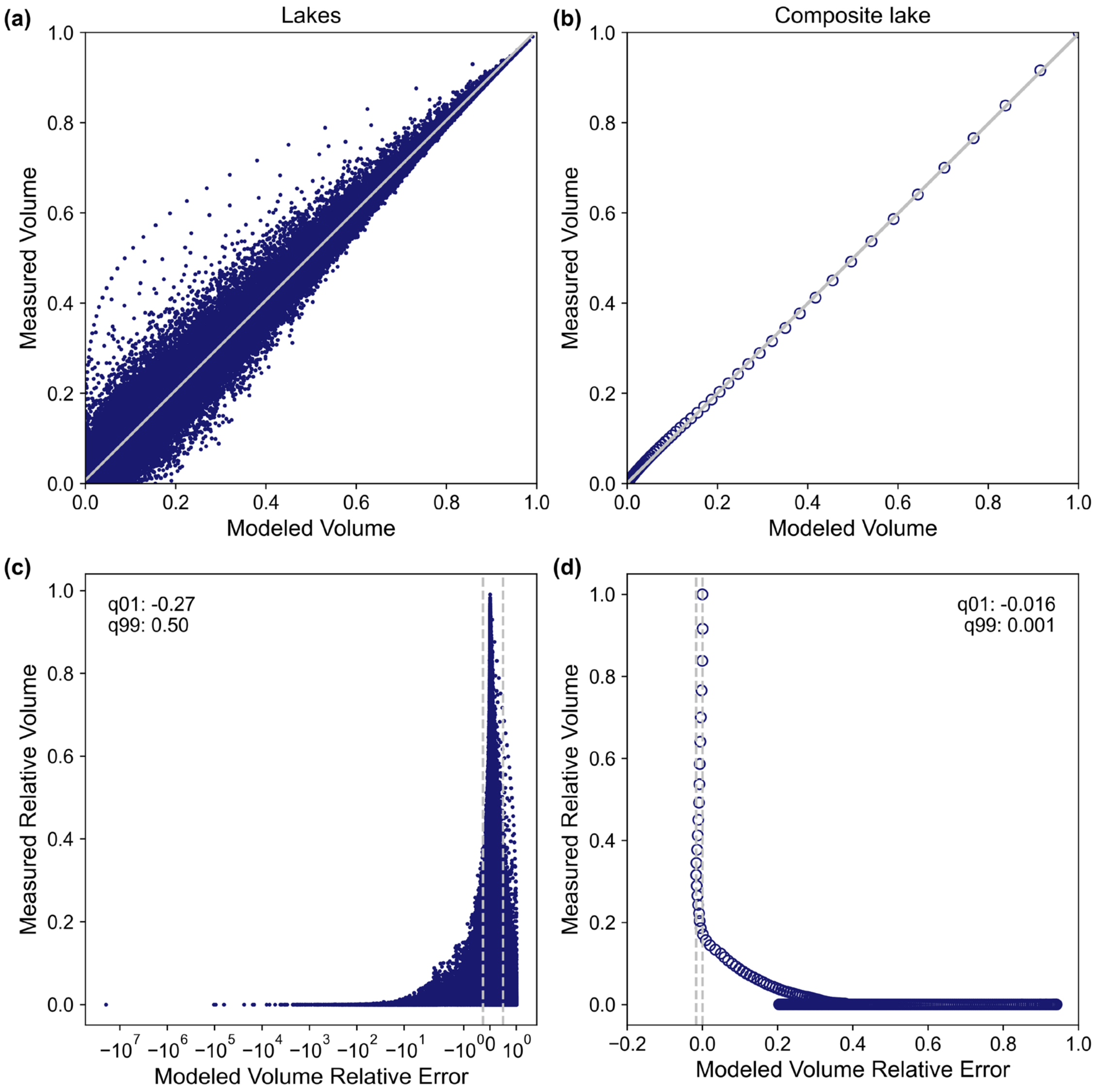

Supplementary Figure S6. The measured and modeled volumes below depth of lakes in Minnesota and Finland. (**a**) The relationship between measured and modeled volumes below depth (Eq. 5, Methods) of individual lakes. (**b**) The aggregated volumes below depth (Eq. 12, Methods) of the composite lake. (**c**) The relative errors for the modeled volume below depth of the individual lakes (symlog scale) and (**d**) composite lake. The red dashed lines mark the 1st and 99th percentiles of the relative areas at depths exceeding 0.2. The summary of the linear model fit in relative and absolute terms for lakes (**a**) and composite lake (**b**) can be found in Supplementary Tables S5 and S6, respectively. Bathymetry data from Minnesota and Finland (Supplementary Table S1).

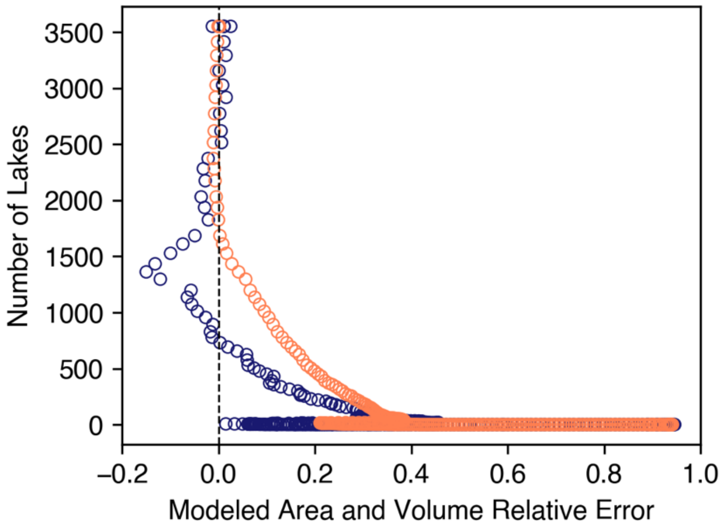

Supplementary Figure S7. The relationship between the number of lakes in the composite and relative error. Lake numbers used in the composite lake aggregation and the relative errors in modeled lake area and volume, are represented by blue and orange circles respectively. Bathymetry data from Minnesota and Finland (Supplementary Table S1).

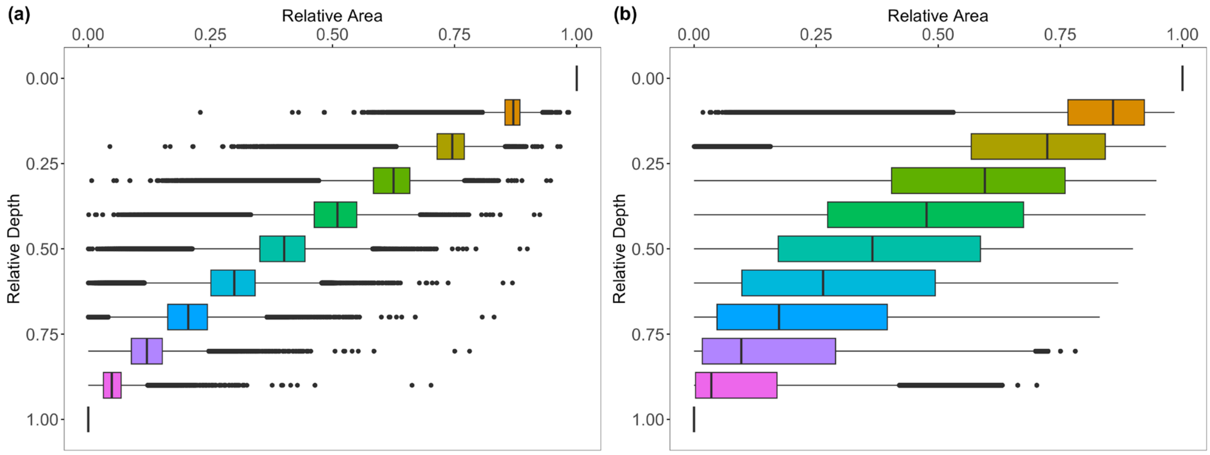

Supplementary Figure S8. Boxplots illustrating the predicted and resampled hypsography of individual lakes. Boxplots illustrating predicted and resampled lake hypsography for 5,735,464 lakes globally. Each plot summarizes relative area values at 11 standard relative depth levels (0.0 to 1.0). (**a**) Predicted hypsographic profiles based on Eq. 2 (Methods), using modeled maximum depth (Z_{max}) and shape parameter (q) for each lake (iteration = 1). (**b**) Resampled hypsographies generated by repeatedly drawing from the distribution of predicted Zmax and q values across all lakes (iterations = 2–6), capturing model validation uncertainty (see Supplementary Table S3). For each relative depth level, boxplots display the distribution of relative area values across all lakes. The center line marks the median (50th percentile), box bounds show the interquartile range (25th–75th percentiles), and whiskers extend to the most extreme values within 1.5× the interquartile range. The y-axis is reversed to indicate increasing relative depth from surface to bottom.

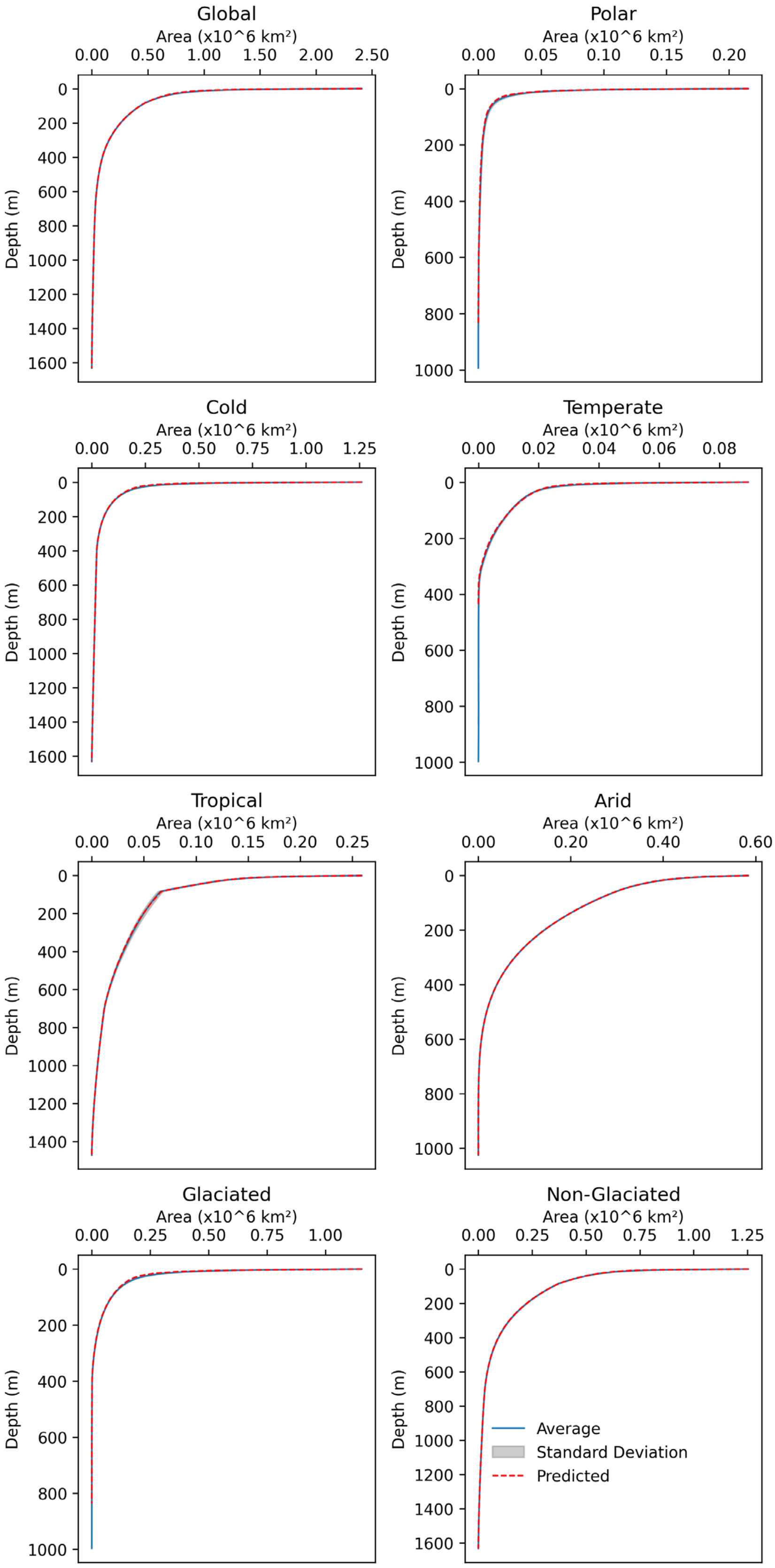

Supplementary Figure S9. Composite lakes variability across global and climate regions. The red dashed line represents the aggregated hypsographies using predicted values of Z_{max} and q. Conversely, the blue line represents the average hypsography derived from 100 iterations of composite lake, each computed by aggregating lake hypsographies modeled with resampled Z_{max} and q through Monte Carlo simulations. The grey band indicates the SD from these 100 iterations.

Supplementary Table S1. Summary of lake characteristics of lakes with measured bathymetry. Data comprising 1871 lakes in Finland and 1908 lakes in Minnesota as well as 39806 lakes with morphometry data.

Morphometry	Unit	Statistics	Finland	Minnesota	MorphoData
Surface Area	m^2	Max	155327556.7	518499955.8	378400000000
Surface Area	m^2	Mean	2371469.3	3185704.4	39560575.6
Surface Area	m^2	Median	584020.1	920309.5	406000
Surface Area	m^2	Min	2314.4	8444.0	1000
Volume	m^3	Max	1405042941.2	4507498328.7	68868800000000
Volume	m^3	Mean	13120410.6	18321918.7	4250578088.1
Volume	m^3	Median	1674470.7	3044238.3	1372511.6
Volume	m^3	Min	2039.2	18611.3	167.5
Z_{max}	m	Max	75.5	141.7	1632
Z_{max}	m	Mean	10.6	11.6	15.6
Z_{max}	m	Median	8.1	9.1	9.7
Z_{max}	m	Min	0.5	0.6	0.2
Z_{mean}	m	Max	23.0	53.1	744.5
Z_{mean}	m	Mean	3.3	4.1	5.8
Z_{mean}	m	Median	2.8	3.5	3.6
Z_{mean}	m	Min	0.2	0.1	0.03

Supplementary Table S2. The data sources and availability.

Source	Web address	N	Dataset	Data availability*
A morphometric atlas of Alaskan lakes: Cook inlet, Prince William Sound, and bristol bay areas. 2000. M. A. Spafard and J. A. Edmundson. Regional Information Report No. 2A00-23. Alaska Department of Fish and Game Commercial Fisheries Division, 333 Raspberry Road, Anchorage, Alaska 99578-1599.	-	247	Morphometry	Public
Adirondack Lakes Survey Corporation	www.adirondacklakessurvey.org	1520	Morphometry	Upon request
Alin, S. R. & Johnson, T. C. Carbon cycling in large lakes of the world: A synthesis of production, burial, and lake-atmosphere exchange estimates. Global Biogeochem. Cycles 21, GB3002–doi:10.1029–2006GB002881. (2007).	-	34	Morphometry	Public
British Columbia Lake Surveys spatial data layer in the BC Provincial Government BC Geographic Data Warehouse of the BC Ministry of Environment	http://www.env.gov.bc.ca.	2788	Morphometry	Upon request
Centre for Ecology & Hydrology (CEH), Edinburgh	http://www.ceh.ac.uk	732	Morphometry	Upon request
Danish lakes included in the Danish River Basin Management Plans (2015-2021). Miljø- og Fødevareministeriet Miljøstyrelsen	www.mst.dk	622	Morphometry	Upon request
Federal Office for the Environment FOEN, Switzerland	www.bafu.admin.ch	109	Morphometry	Upon request

Source	Web address	N	Dataset	Data availability*
Finnish Environment Institute (SYKE)	https://www.syke.fi/en-US	5557	Morphometry	Upon request
Global Lake Data Base (GLDB)	http://www.flake.igb-berlin.de/ep-data.shtml (GLDB, 2019).	3046	Morphometry	Public
Maine Dept. Environmental Protection, Maine Dept. Inland Fisheries & Wildlife, Maine Office of GIS, Augusta	http://www.gulfofmaine.org/kb/files/9680/MaineLakes_Geography_Morphometry.xls	1871	Morphometry	Public
Michigan Department of Natural Resources Institute for Fisheries Research	https://www.michigan.gov/dnr/0,4570,7-350-79136_79236_80537_82192---,00.html	2198	Morphometry	Upon request
Minnesota DNR, Division of Fish and Wildlife	https://gisdata.mn.gov/dataset/water-lake-basin-morphology	1933	Morphometry	Public
Mosquera, P.V., Hampel, H., Vázquez, R.F., Alonso, M., Catalan, J. 2017. Abundance and morphometry changes across the high mountain lake-size gradient in the tropical Andes of Southern Ecuador. Water Resources Research, doi: 10.1002/2017WR020902	-	170	Morphometry	Public
New Hampshire Department of Environmental Services	www.des.nh.gov	690	Morphometry	Upon request
Nova Scotia Environment	https://novascotia.ca/nse/Default.asp	986	Morphometry	Upon request
Ontario Ministry of Northern Development, Mines, Natural Resources and Forestry	https://www.ontario.ca/page/ministry-northern-development-mines-natural-resources-forestry	9740	Morphometry	Upon request
Portland State University, Environmental Science and Management, Center for Lakes and Reservoirs	https://pdxscholar.library.pdx.edu/centerforlakes/	152	Morphometry	Upon request
Quebec, present study	-	444	Morphometry	Upon request
Swedish lake register. Swedish Hydrological and Meteorological Institute	www. smhi.se	4983	Morphometry	Public
Water Institute, University of South Florida	www.waterinstitute.usf.edu	354	Morphometry	Upon request
Wisconsin Department of Natural Resources, USA	http://dnr.wi.gov/	1632	Morphometry	Upon request
Finnish Environment Institute (SYKE)	https://www.syke.fi/en-US/Open_information/Spatial_datasets/Downloadable_spatial_dataset#L	1871	Bathymetry	Public
Minnesota DNR, Division of Fish and Wildlife	https://gisdata.mn.gov/dataset/water-lake-basin-morphology	1908	Bathymetry	Public
Swiss Federal Institute of Aquatic Science and Technology (EAWAG)	https://www.eawag.ch/en/	34	Bathymetry	Upon request

* Data is public if it can be directly accessed at the organization website or through a publication, otherwise data was made available to the authors upon request and can be requested at the source.

Supplementary Table S3. Summary of the random forest models goodness of fit for the prediction of Z_{mean}, Z_{max} and *q*. The fit parameters are reported for transformed data of power exponent of 0.33. N = number of lakes included in the analysis.

	model	R^2	RMSE	N
Large lakes (>10 km2)				
Z_{mean}	Training	0.914	0.297	1557
	Validation	0.768	0.518	342
Z_{max}	Training	0.900	0.421	1557
	Validation	0.732	0.738	342
q	Training	0.764	0.259	1557
	Validation	0.314	0.450	342
Small lakes (<10 km2)				
Z_{mean}	Training	0.574	0.273	26538
	Validation	0.424	0.308	6681
Z_{max}	Training	0.573	0.401	26538
	Validation	0.428	0.450	6681
q	Training	0.334	0.308	26536
	Validation	0.115	0.350	6680

Supplementary Table S4. The one-degree composite lake clusters and their morphometry parameters. Total number of one-degree grids per cluster (nGrids). The statistics of the one-degree composite lakes morphometry parameters reflect median composite lakes parameter values per cluster. Number of lakes per grid (nLakes)*, Surface Area (SA_C), maximum depth (Z_{maxC}), mean depth (Z_{meanC}), fraction of epilimnetic volume (fV_{EpiC}), fraction of epilimnetic sediment surface area (fA_{EpiC}), epilimnetic volume and area (V_{EpiC}/A_{EpiC}), dynamic ratio (DR_C).

Composite lake clusters morphometry	Cluster 1	Cluster 2	Cluster 3	Cluster 4	Cluster 5
nGrids	4021	2619	7753	82	2
nLakes	80	1023	33	89.5	11.5
SA_C (km^2)	9.5	214.5	4.4	340.2	32504.8
Z_{maxC} (m)	25.1	17	9.7	306.0	1551.5
Z_{meanC} (m)	4.9	2.8	2.8	105.8	658.5
V_C (km^3)	0.05	0.62	0.01	32.5	21370.5
fV_{EpiC}	0.25	0.31	0.80	0.27	0.25
fA_{EpiC}	0.23	0.29	0.74	0.31	0.25
V_{EpiC}/A_{EpiC} (m^2/m^3)	5.7	3.0	3.0	108.1	673.0
DR_C	0.5	4.7	0.6	0.2	0.3

*In some cases, large lakes can cross multiple grids. For such cases, the lake centroid was attributed only to one grid.

Supplementary Table S5. Results of the relative measured and modeled linear models. Table describes the relationship between observed and modeled relative areas and volumes below depth.

	slope	intercept	R^2	DF	F Ratio	RMSE	Prob>|t| slope
Fig. S5a	0.9871386	0.0055917	0.977	(1, 89394)	3921400	0.047851	<0.0001*
Fig. S5b	1.0050355	-0.000137	0.999	(1, 282)	525149	0.003298	<0.0001*
Fig. S6a	0.9966531	0.0074355	0.988	(1, 89394)	7337073	0.033318	<0.0001*
Fig. S6b	0.9958518	0.0010661	0.999	(1, 282)	860374	0.002564	<0.0001*

Supplementary Table S6. Results of the absolute measured and modeled linear models. Table describe the relationship between observed and modeled absolute areas at depth and volumes below depth. We used m^2 for area and m^3 for volume.

	slope	intercept	R^2	DF	F Ratio	RMSE	Prob>|t| slope
Fig. S5a	1.0323337	-42433.4	0.983	(1, 89394)	4836900	1172854	<0.0001*
Fig. S5b	1.0049727	-1435569	0.999	(1, 282)	525149	34672858	<0.0001*
Fig. S6a	0.9895501	267535.2	0.990	(1, 89394)	11798417	4660195	<0.0001*
Fig. S6b	0.9958172	63477968	0.999	(1, 282)	860374	152652788	<0.0001*

Supplementary Table S7. The global and regional composite lake morphometry parameters. The mean and SD of DR_C, Z_{maxC}, Z_{meanC} calculated based on 100 resampled iterations. The SD of V_C, vas calculated based on the integrated SD (Eq. 1, Supplementary Methods) as well as the average of the V_C based on 100 iteration of Monte Carlo simulations in the brackets. For details on the SD calculations for each parameters see Supplementary Methods section. N-G in this table refers to the Nonglaciated region.

Parameter	Global	Glaciated	N-G	Polar	Cold	Temperate	Arid	Tropical
DR_C	22.7	42.9	10.3	28.3	27.4	8.8	6.1	3.9
DR_C SD	0.12	0.45	0.06	1.40	0.16	0.20	0.0	0.09
Z_{maxC} (m)	1632	850.6	1632	852.5	1632	584	1025	1471
Z_{maxC} SD	0.0	33.9	0.0	36.5	0.0	87.7	0.0	0.0
Z_{meanC} (m)	68.5	42.9	108.5	16.4	41.0	33.9	125.0	130.2
Z_{meanC} SD	0.35	0.27	0.61	0.83	0.24	0.77	0.01	3.0
V_C (km^3)	164993	286958	136035	3534	51668	3038	73021	33733
V_C SD	10137	1709	9975	1798	1607	887	371	10051
	(852)	(307)	(770)	(178)	(305)	(69)	(73)	(767)
V_{EpiC} (km^3)	70947	14768	55774	1343	22734	1337	37241	7759
V_{EpiC} SD	4359	872	4090	683	707	390	189	2312
SA_C (km^2)	2408550	1154794	1253756	215321	1260198	89588	584358	259085
SA_{zC} SD*	62618	45392	42923	18623	43959	6763	11427	39823
A_{EpiC} (km^2)	1035676	392630	639416	45217	441069	63607	321397	152860
A_{EpiC} SD	26926	15433	21891	3911	15386	4802	6285	23495
V_{EpiC} /A_{EpiC} (m^3 m^{-2})	68.5	37.6	87.2	29.7	51.5	21.0	115.9	50.8
V_{EpiC} /A_{EpiC} SD	4.6	2.7	7.1	15.3	2.4	6.4	2.3	18.1

*SD of area at depth

References

1 Pekel, J. F., Cottam, A., Gorelick, N. & Belward, A. S. High-resolution mapping of global surface water and its long-term changes. *Nature* **540**, 418-422 (2016).

2 Verpoorter, C., Kutser, T., Seekell, D. A. & Tranvik, L. J. A global inventory of lakes based on high-resolution satellite imagery. *Geophys. Res. Lett.* **41**, 6396-6402 (2014).

3 Feng, M., Sexton, J. O., Channan, S. & Townshend, J. R. A global, high-resolution (30-m) inland water body dataset for 2000: first results of a topographic–spectral classification algorithm. *Int. J. Digit. Earth* **9**, 113-133 (2015).

4 Allen, G. H. & Pavelsky, T. M. Global extent of rivers and streams. *Science* **361**, 585-588 (2018).

5 Lehner, B. *et al.* High-resolution mapping of the world's reservoirs and dams for sustainable river-flow management. *Front. Ecol. Environ.* **9**, 494-502 (2011).

6 Gorelick, N. *et al.* Google Earth Engine: Planetary-scale geospatial analysis for everyone. *Remote Sens. Environ.* **202**, 18-27 (2017).

7 QGIS Development Team. *QGIS Geographic Information System*. Open Source Geospatial Foundation Project. URL http://qgis.org.
8 PostGIS Development Team. PostGIS: Spatial and geographic objects for PostgreSQL. https://postgis.net/ (2023). Open Source Geospatial Foundation.
9 Messager, M. L., Lehner, B., Grill, G., Nedeva, I. & Schmitt, O. Estimating the volume and age of water stored in global lakes using a geo-statistical approach. *Nat. Commun.* **7**, 13603 (2016).
10 Finnish Environment Institute (SYKE). *Lake and river depth datasets: Sounding points (Luotauspisteet) and depth areas (Syvyysalue)*. Shapefiles, CC BY 4.0. Finnish Environment Institute (2025). https://www.syke.fi/en/environmental-data/downloadable-spatial-datasets#lake-and-river-depth.
11 Minnesota Department of Natural Resources, Division of Fish & Wildlife – Section of Fisheries. *Lake Bathymetric Outlines, Contours, and DEM*. Minnesota Department of Natural Resources (2024). https://gisdata.mn.gov/dataset/water-lake-bathymetry.
12 Lucas, G. Tinfour: high-performance 2D Delaunay triangulation software written in java (version 2.1.8) (2024). [Computer software] https://github.com/gwlucastrig/Tinfour.
13 Muñoz Sabater, J. ERA5-Land hourly data from 1950 to present. (Copernicus Climate Change Service (C3S) Climate Data Store (CDS). Accessed on 2022-03-06 and 2022-03-07, 2019).
14 Hondzo, M. & Stefan, H. G. Lake water temperature simulation model. *J. Hydraul. Eng.* **119**, 1251-1273 (1993).
15 Woolway, R. I. & Merchant, C. J. Worldwide alteration of lake mixing regimes in response to climate change. *Nat. Geosci.* **12**, 271-276 (2019).
16 Maberly, S. C. *et al.* Global lake thermal regions shift under climate change. *Nat. Commun.* **11**, 1232 (2020).
17 Wetzel, R. G. *Limnology*. 2nd edn, (Saunders College Publishing, 1983).
18 Soranno, P. A., Lottig, N. R., Delany, A. D. & Cheruvelil, K. S. (2019).
19 Grant, L. *et al.* Attribution of global lake systems change to anthropogenic forcing. *Nat. Geosci.* **14**, 849-854 (2021).
20 Håkanson, L. Models to predict Secchi depth in small glacial lakes. *Aquat. Sci.* **57**, 31-53 (1995).
21 Goudsmit, G. H., Burchard, H., Peeters, F. & Wüest, A. Application of k-ϵ turbulence models to enclosed basins: The role of internal seiches. *J. Geophys. Res. Oceans* **107**, 3230-3242 (2002).
22 Råman Vinnå, L., Medhaug, I., Schmid, M. & Bouffard, D. The vulnerability of lakes to climate change along an altitudinal gradient. *Commun. Earth Environ.* **2**, 2-35 (2021).
23 Thiery, W. *et al.* LakeMIP Kivu: evaluating the representation of a large, deep tropical lake by a set of one-dimensional lake models. *Tellus A: Dyn. Meteorol. Oceanogr.* **66** (2014).
24 Woolway, R. I. *et al.* Phenological shifts in lake stratification under climate change. *Nat. Commun.* **12**, 2318 (2021).
25 Zhuang, Q. *et al.* Current and Future Global Lake Methane Emissions: A Process-Based Modeling Analysis. *J. Geophys. Res. Biogeosci.* **128** (2023).
26 Golub, M. *et al.* A framework for ensemble modelling of climate change impacts on lakes worldwide: the ISIMIP Lake Sector. *Geosci. Model Dev.* **15**, 4597-4623 (2022).
27 Guo, M. *et al.* Intercomparison of Thermal Regime Algorithms in 1-D Lake Models. *Water Resour. Res.* **57**, 1-21 (2021).
28 Plummer, M., Best, N., Cowles, K. & Vines, K. CODA: Convergence Diagnosis and Output Analysis for MCMC. *R News* **6**, 7-11 (2006).

29 Hollister, J. W., Milstead, W. B. & Urrutia, M. A. Predicting maximum lake depth from surrounding topography. *PLoS One* **6**, e25764 (2011).
30 Minns, C. K., Moore, J. E., Shuter, B. J. & Mandrak, N. E. A preliminary national analysis of some key characteristics of Canadian lakes. *Can. J. Fish. Aquat. Sci.* **65**, 1763-1778 (2008).
31 Heathcote, A. J., del Giorgio, P. A., Prairie, Y. T. & Brickman, D. Predicting bathymetric features of lakes from the topography of their surrounding landscape. *Can. J. Fish. Aquat. Sci.* **72**, 643-650 (2015).
32 Oliver, S. K. *et al.* Prediction of lake depth across a 17-state region in the United States. *Inland Waters* **6**, 314-324 (2016).
33 Sobek, S. Predicting the depth and volume of lakes from map-derived parameters. *Inland Waters* **1**, 177-184 (2011).
34 Cael, B. B., Heathcote, A. J. & Seekell, D. A. The volume and mean depth of Earth's lakes. *Geophys. Res. Lett.* **44**, 209-218 (2017).
35 Meybeck, M., Green, P. & Vorosmarty, C. A new typology for mountains and other relief classes: An application to global continental water resources and population distribution. *Mt. Res. Dev.* **21**, 34-45 (2001).
36 European Soil Data Centre (ESDAC). Global landform classification (GIS raster dataset) (2020). URL https://esdac.jrc.ec.europa.eu/content/global-landform-c lassif ication. Accessed: 2020-09-06.
